# The role of fear learning in the development of psychosis: an EEG study utilizing a differential fear conditioning paradigm in people with psychotic vulnerability

**DOI:** 10.1038/s41537-026-00761-y

**Published:** 2026-05-15

**Authors:** Metin Özyagcilar, Nilay Esin Ahrens-Demirdal, Anja Riesel, Tina B. Lonsdorf, Tania M. Lincoln

**Affiliations:** 1 Clinical Psychology and Psychotherapy, Institute of Psychology, Faculty of Psychology and Human Movement Science, Universität Hamburg, Hamburg, Germany; 2 Clinical Psychology and Neuroscience, Institute of Psychology, Faculty of Psychology and Human Movement Science, Universität Hamburg, Hamburg, Germany; 3 Biological Psychology and Cognitive Neuroscience, Department of Psychology, Universität Bielefeld, Bielefeld, Germany; 4 Institute of Systems Neuroscience, University Medical Center Hamburg-Eppendorf, Hamburg, Germany

**Keywords:** Human behaviour, Biomarkers

## Abstract

By studying how individuals in an “at-risk” state of psychosis learn about threat and safety cues – specifically, how they develop and unlearn fear responses to neutral cues – we might better understand the mechanisms leading to heightened arousal and fear that are characteristic of acute psychotic episodes. At-risk individuals (*N* = 88; of which 28 fulfilled ultra-high-risk criteria on the Comprehensive Assessment of At-Risk Mental States interview and 60 scored above a predefined threshold on the Community Assessment of Psychic Experiences) and healthy controls (*N* = 44) underwent a standardized and validated differential fear conditioning paradigm including an acquisition, generalization, and extinction phase. The main outcomes of interest were the late positive potential, fear-potentiated startle, and self-reported ratings of valence, arousal, fear, and expectancy elicited by the conditioned stimuli (CS). The at-risk group exhibited diminished fear learning, evident in significantly reduced differentiation between the CS+ vs. CS- in the valence ratings compared to controls. Additionally, they demonstrated impaired fear extinction, evident in valence and arousal ratings, in which their CS differentiation showed a slower reduction than the controls. There were no group differences in late positive potential responses. At risk mental states appear to be associated with problems in distinguishing dangerous from safe stimuli and a diminished ability to adjust affective responses to conditioned stimuli based on new information, while the late-positive potential and fear-potentiated startle are unaltered. Early interventions could focus on recalibrating subjective emotional evaluations of fear-associated events.

## Introduction

The classic fear conditioning paradigm has provided a powerful model of emotional learning and forms the foundation of the exposure treatments for anxiety disorders^1^. Fear conditioning paradigms involve pairing a neutral stimulus (CS) with an aversive one (US), leading to a conditioned response (CR) that can be reversed through extinction^2^. In this context, high reactivity to the CS- (i.e., the stimulus that is not paired with the US), resulting in reduced differentiation of the CS+ (i.e., paired with the US) from the CS-, is to be a form of maladaptive fear learning and contributes to the development of anxiety disorders^3^.

Here, we argue that aberrant fear learning should also be relevant to the development of psychotic symptoms. Anxiety symptoms co-exist with psychotic disorders^4^ and positive symptoms of psychosis, such as paranoid delusions^5^. Psychosis shares several core features with anxiety disorders. These include heightened psychophysiological arousal^6–8^ and high levels of avoidance^9^. Also, people with psychosis have been found to misattribute salience to neutral faces^10,11^, and aberrations in the attribution of neutral faces in individuals at increased risk for psychosis have even been found to predict subsequent transition to full psychosis^12^. According to the aberrant salience theory^13^, people with psychosis assign significance to neutral stimuli, which could manifest as increased CS- sensitivity in fear conditioning.

The CR can be measured via self-reports, physiological responses, or neural activity^14^. Given their high temporal resolution, and the fact that they are less prone to habituation than autonomic responses and are less affected by the biases present in self reports, using event related potentials, such as the late positive potential (LPP) via electroencephalogram (EEG) has become increasingly common^15–19^. The LPP can be measured over parietal areas and is thought to index motivated attention to “significant” stimuli^20^. It is therefore a suitable event-related-potential candidate to capture the complex higher-order attentional mechanisms, particularly dysregulated attribution of salience to events that are observed in psychosis.

So far, differential fear conditioning paradigms have rarely been used to study psychosis. The few that have, largely find patients with psychosis to show diminished differentiation between the CSs during acquisition^21–25^ and diminished extinction memory^21^. To our knowledge, only one study investigated generalization and contradictorily reported under-generalization in patients^26^. A couple of studies also investigated whether these aberrancies are evident prior to a formal diagnosis. Young adults at risk of developing psychosis demonstrated impaired fear acquisition, evident in a reduced LPP effect (i.e., amplitude difference in response to CS+ vs. CS-)^27^. Using social stimuli (i.e., faces) as the CSs, another study reported reduced differential ventromedial prefrontal cortex activation (i.e., safety responding; CS- vs CS+) in individuals with a clinical high-risk (CHR) status as compared to controls^24^. Using a similar paradigm, other researchers^28,29^ found participants with high versus low delusion proneness (during non-instructed fear learning) to show impaired fear extinction learning across friendliness ratings, skin conductance responses, and fMRI measures, including medial prefrontal activity. In contrast, no group differences during acquisition were observed in friendliness ratings, SCR, or fMRI measures. Taken together, the sparse existing research indicates psychosis to be related to impairments in fear acquisition and extinction. However, the studies conducted in at-risk groups have yielded mixed findings and are difficult to interpret due to the isolated examination of specific phases of fear learning or outcomes.

To investigate potential aberrations in different phases of fear learning in individuals at-risk of developing psychosis, our study employs a well-established differential fear conditioning paradigm that integrates multiple phases of fear learning, including acquisition, generalization, and extinction, and uses neutral stimuli as CSs. This is combined with a comprehensive set of behavioral (i.e., self-report ratings: expectancy, valence, arousal, and fear) and neurophysiological measures (i.e., fear-potentiated startle, FPS; and LPP). Based on the theoretical framework and existing research, we predicted that the at-risk group would exhibit diminished fear acquisition learning (i.e., a smaller discrimination in LPP response, FPS, and self-report ratings between CS+ vs CS-) and diminished fear extinction learning (i.e., the discrimination in the responses to CS+ vs. CS- showing a weaker decline from the first until the last block of the extinction phase for LPPs and from the generalization to the extinction phase for FPS and self-report ratings) compared to the healthy controls (HC). Also, given the scarcity of research in fear generalization, the present study sought to address this gap by conducting an exploratory analysis of group differences.

## Methods and materials

### Pre-registration

The hypotheses are pre-registered on Open Science Framework platform along with further methodological details, including additional assessments and exclusion criteria: https://osf.io/2dgtc. Any deviations from pre-registration are explicitly noted.

### Procedure and participants

The procedure started with an online survey using the German version of the Community Assessment of Psychic Experiences (CAPE, positive dimension)^30,31^ to identify participants with scores ≥2.8 and thus at an increased risk of psychosis^32^. The survey also included questions on demographics and on potential exclusion criteria for EEG. Eligible participants were contacted by phone and screened for acute and past mental disorders using the Structured Clinical Interview for the Diagnostic and Statistical Manual of Mental Disorders - 5 (Clinical Version)^33^. In the following on-site assessment, participants who scored above the CAPE cut-off were interviewed with the Comprehensive Assessment of At-Risk Mental States (CAARMS)^34^ to determine whether they fulfilled criteria for a CHR status. Those who did not fulfill the criteria were assigned to the psychosis proneness (PP) group.

Screening data were collected online from a total of *N* = 2224 participants, aged between 18 and 65 years old. Of the 132 participants included in the study, 28 were allocated to the CHR, 60 to the PP, 44 to the HC group. An exclusion criterium for the PP and CHR groups was a current or lifetime diagnosis of a psychotic disorder. For the HC group any current or previous mental health diagnosis and usage of psychotropic medication were exclusion criteria.

After the clinical assessment, a baseline resting-state EEG and electrocardiogram were recorded. Subsequently, participants completed the differential classical fear conditioning paradigm. Participants were offered either a monetary or a non-monetary (i.e., partial credits, applicable for psychology students) compensation for their participation.

### Measures

The CAPE^30,31^ covers three symptom dimensions of psychosis (positive, negative, depression), of which the positive subscale (20 items) was used for this study. Each item is scored on frequency and distress on 4-point Likert scales. The time frame of the items was modified from life-time to “during the last 4 weeks” to capture current symptomatology. In this study, the positive CAPE subscale demonstrated high internal consistency, as indicated by a Cronbach’s alpha of *α* = 0.91.

The CAARMS^34^ is a semi-structured clinical interview. The Positive Symptom Scale of the CAARMS includes the subscales Unusual Thought Content, Non-Bizarre Ideas, Perceptual Abnormalities, and Disorganized Speech rated based on intensity, which ranges from 0 (“never, absent”) to 6 (“psychotic and severe”), and frequency, which ranges from 0 (“absent”) to 6 (“continuous”). The Social and Occupational Functioning Scale of the CAARMS assesses social and occupational functioning ranging from 0 to 100. The combined information from these scales is used to determine whether individuals meet criteria for CHR (for information on scoring and classification, see ref. ^35^). Out of 28 participants fulfilling CHR criteria, 25 were allocated to the attenuated psychosis group, 2 to the vulnerability group, 0 to the brief limited intermittent psychotic symptoms group, and 1 participant remained unclassified due to missing data.

### The differential fear conditioning paradigm

**Fig. 1:**
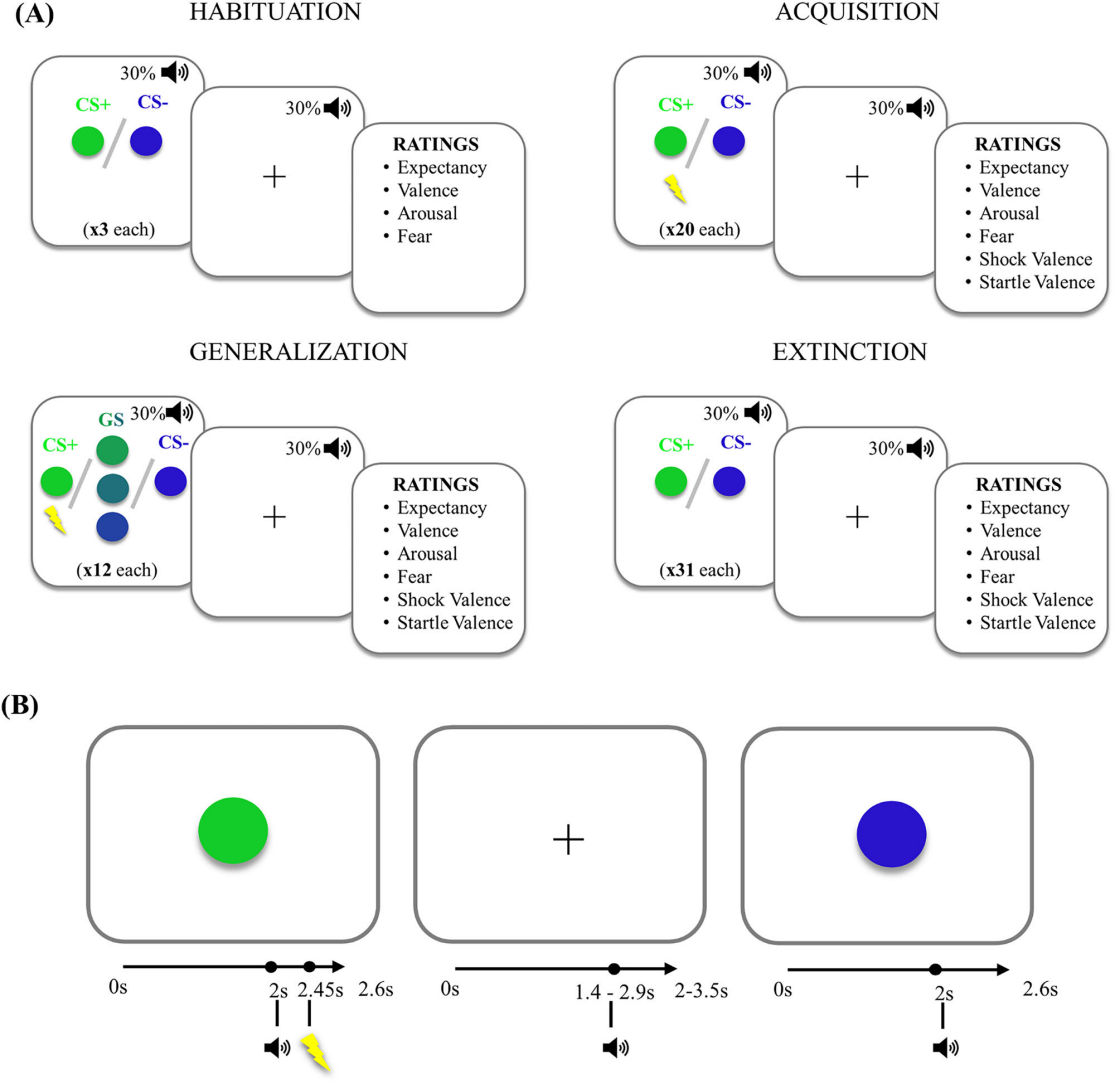
The differential fear conditioning paradigm. **A** depicts the five phases of the paradigm and **B** the trial order, including durations of the stimuli. The green circle is depicted here as conditioned stimuli (CS+), the unconditioned stimuli (US) is depicted with the lightning and startle with the sound symbol. The colors of the CS+ were counterbalanced across the participants. All ratings were presented on a continuous scale with scores ranging from 0 to 100. Inter-trial-interval duration varied between 2–3.5 s.

The paradigm is depicted in Fig. 1.

Five circles with five different colors that varied along a blue to green dimension were used as CSs. The CS+ and CS- were the circles that were the most blue or the most green. The three intermediate stimuli were used as generalization stimuli (GS): GS+ was most similar to the CS+, GS- to the CS- and GSU was ambiguous. Electro-tactile stimulation served as the unconditioned stimuli (US) and was, delivered only after CS+ trials. It was administered on the back side of the dominant hand and was calibrated to be unpleasant but not painful^14^. For the US, the reinforcement rate was set to 80% during the fear acquisition and generalization phase. Prior to the experiment, participants were told that one of the circle presentations would be followed by an electro-tactile stimulation, but they were not informed about the CS contingencies.

The CS was always presented for 2600 ms, with an inter-trial interval (ITI) of 2000–3500 ms. The US was delivered after 2450 ms of the CS onset, terminating with the CS offset. The startle sound was delivered 600 ms before CS or ITI offset.

Before the habituation phase, participants were informed that no US administration would take place during that phase. During the habituation phase, prior to the CS presentation, the startle sound was delivered ten times alone^36^, which consisted of 95 dB bursts of white noise (50 ms, rise/fall time <1 ms) and delivered binaurally through headphones. This was followed by the presentation of the CS+ and the CS- three times each. Before the acquisition phase, participants were informed that US administration could take place at any point. During acquisition, the CS+ and the CS- were presented 20 times each. Subsequently, two CSs as well as the three GSs were presented 12 times each during the fear generalization phase. The fear extinction phase followed, during which the CS+ and the CS- were presented 31 times each. Between phases, participants were instructed to rate the level of US expectancy, valence (i.e., unpleasantness), arousal, and fear associated with each type of CS, the level of unpleasantness of the US and startle sound on visual analog scales (with anchors “0”: “not at all” and “100”: “extremely”). Finally, all stimuli were displayed on the screen and participants were asked to select the ones that had been followed by the US. This question was correctly answered by 97.7% of the participants.

### EEG and FPS data acquisition and processing

EEG was recorded using 64 sintered Ag/AgCl active electrodes positioned on an equidistant cap (EasyCap GmbH) and an actiCHamp Plus amplifier (Brain Products GmbH). Data was sampled at 1000 Hz and filtered online (DC–280 Hz). The reference electrode was placed between AF3 and Fz, the ground between AF4 and Fz. Vertical eye movements were monitored via an external electrode below the left eye. Preprocessing was conducted in MATLAB Version 2023b,^37^ using EEGLAB Version 2023.1^38^, applying a low- and high-pass finite impulse response filter with a higher edge of 30 Hz, a lower edge of 0.01 Hz. 50 Hz line noise removal was applied as in the PREP pipeline^39^. Noisy channels were visually identified and interpolated using spherical spline interpolation. Independent Component Analysis was performed to correct for eye blink and saccade artifacts, with component selection based on scalp maps, time courses, and power spectra. Cleaned data was re-referenced to the average of all scalp electrodes^40,41^, and segmented from −400 to 2600 ms around stimulus onset. Remaining artifacts were rejected using FASTER^42^ (>3 z-scores in amplitude, variance, or channel deviation). Trial counts remaining after artifact rejection are detailed in Table S2. Baseline correction was applied (−200 to 0 ms). Upon visual inspection, LPP amplitudes were averaged over PO1, PO2, Pz, O1, O2, and Oz^19,43^ in early (300–1000 ms) and late (1000–2000 ms) windows^43,44^.

FPS was recorded via two Ag/AgCl electrodes placed below the right eyelid (1000 Hz sampling rate). A ground electrode was placed on the right infraorbital triangle. Data was pre-processed in the Brain Vision Analyzer^45^, using filtering (28–500 Hz), rectification, baseline-correction, and integration. Startle responses (i.e., peak) were analyzed in a 20–150 ms post-probe window. Participants with >66% invalid trials were excluded from FPS analyses (*n* = 25). Further details are provided in Supplement 1.

### Statistical analyses

As pre-registered, the CHR and PP were combined to the “at-risk” group (*N* = 88) for hypothesis testing.

Group differences in baseline variables were tested with *t*-tests for continuous variables and with chi-square tests (*χ*2) for categorical variables. All hypotheses were tested with linear mixed-effects models implemented in the R Software^46^. Pairwise comparisons were conducted to follow up significant effects, using the emmeans package^47^ with the Tukey method to adjust for multiple comparisons. Satterthwaite approximations for degrees of freedom were used to compute *p* values. Separate models were calculated for each of the dependent variables (i.e., ratings, LPPs, FPS) for each phase.

As a manipulation check, we tested that (1) successful fear acquisition, (2) gradual fear generalization, and (3) successful fear extinction would be demonstrated in the complete sample using three separate models which included the fixed effect CS type. The model testing extinction also included a fixed effect of block (first block, final block) when the LPP were analyzed as outcome and of phase (generalization vs. extinction) when the subjective ratings were analyzed as the outcome; including a two-way interaction with CS type.

The main hypotheses on group differences in acquisition and extinction learning were tested with two separate models (referred to as acquisition model and extinction model in the following). Both of these models included the interaction term CS type x group (At-risk vs. HC). The extinction model also included an additional fixed effect of block (first block: including the first 10 trials; final block: including the last 10 trials) for the LPP, and of phase (generalization vs. extinction) for the subjective ratings, thereby creating the interaction term CS type x group x block/phase. Even though the extinction phase comprised 31 trials per stimulus, the block factor for the LPP analysis was defined using only the first 10 and the last 10 extinction trials in order to capture the early and late stages of extinction. For the fixed effect CS Type, “CS-”; for the fixed effect group, “HC”, for the fixed effect block “first block” and for the fixed effect phase, “generalization” were coded as the reference levels. All of the models included a random intercept by participants and models including the LPP also included a random intercept by electrode^48^. Aligning with the recommendations^49^, we aimed to fit a maximal random effects structure and therefore included all random slopes for within-subject factors.

#### Changes to the protocol as registered

(i) The time-window for the peak detection for the FPS was updated from the pre-registered interval 25–150 ms to 20–150 ms and the interval for the onset detection (i.e., 20–120 ms) was determined post pre-registration. (ii) For extinction, as the amount of startle probes were not equally distributed between the CSs within the blocks consisting of 10 CS trials we conducted phase-wise analyses rather than the pre-registered block-wise analyses. (iii) Due to recruitment challenges in the CHR group, we deviated from the pre-registered target of *n* = 44 per group by continuing to recruit more participants into the PP group (i.e., additional *n* = 16), ensuring we met the overall pre-registered sample size (*n* = 132) for sufficient power (see https://osf.io/2dgtc). (iv) Although a Fear-reinstatement/Return-of-fear phase and skin conductance responses (SCRs) were included in the paradigm, they were not the focus of the study and will not be reported here.

## Results

### Sample characteristics

There were no significant group differences in age, gender, or education between the at-risk and HC group. As expected, CAPE positive scores were significantly higher in the at-risk group. See Table 1 for descriptive statistics and inferential test results.

**Table 1:** Demographic and clinical variables by group.

Variable	At-risk group	Healthy controls	Test statistic
	*n* (%)	*n* (%)	
Gender ^a^			*χ*²(2) = 2.51, *p* = 0.285
men	24 (27.27%%)	8 (18.18%)	
women	62 (70.45%)	36 (81.82%)	
diverse	2 (2.27%)	—	
Education ^b^			*χ*²(7) = 6.53, *p* = 0.48
low	6 (6.82%)	—	
medium	65 (73.86%)	37 (84.09%)	
high	17 (9.32%)	7 (15.91%)	
Diagnoses^c^			
Affective disorders^d^	46 (52.3%)	—	
Anxiety disorders^d^	23 (26.1%)	—	
ADHD	14 (15.9%)	—	
Substance use	6 (6.8%)	—	
PTSD	7 (8.0%)	—	
OCD	4 (4.5%)	—	
	(*M* ± *SD*)	(*M* ± *SD*)	
Age	25.24 ± 6.96	25.30 ± 8.66	t(71.6) = −0.04, *p* = 0.96
CAPE^e^	3.35 ± 0.54	2.23 ± 0.20	t(123.2) = 17.11, *p* < 0.001

*ADHD* attention deficit hyperactivity disorder, *PTSD* post traumatic stress disorder, *OCD* obsessive-compulsive disorder.

^a^Trans individuals are categorized according to their self-identified gender (i.e., men, women or diverse).

^b^Education levels: low: lower secondary education or less (lower than “Gymnasium (Abitur)”), medium; upper secondary education, vocational training, or university degree in applied sciences degree (“Gymnasium (Abitur)”, “Ausbildung” or “Fachhochschulabschluss”), high: university degree (Diplom/Master/Promotion).

^c^Diagnoses include all Axis-1 disorders (current/past) defined in the Structured Clinical Interview for Diagnostic and Statistical Manual of Mental Disorder - 5 (Clinical Version) interview.

^d^Affective: bipolar disorder, depression/anxiety: panic, agoraphobia, social anxiety disorder, generalized anxiety disorder.

^e^Community Assessment of Psychic Experiences (CAPE) positive subscale scores range from 2 to 8.

### Manipulation check—differential fear acquisition, generalization and extinction learning across all participants

Across all subjects, successful fear acquisition (i.e., CS+ > CS-) and fear extinction (i.e., decrease of CS+/CS- difference) were evident in all subjective ratings (all *p*s < 0.001). For FPS, significant responses were absent during the acquisition phase (*p* = 0.11), but became apparent during generalization (*p* < 0.001) and successful fear extinction was also observed (*p* < 0.001) (Fig. S2). Acquisition was not observed in the LPPs across all trials, however, it was evident during the 2nd block (i.e., the last 10 trials) of acquisition in early LPPs (*p* = 0.02), as well as during the generalization (i.e., CS+ > CS-, *p* < 0.001) (see Fig. S1). An extinction effect was also observed in LPPs (all *p*s < 0.001) (see Fig. S1). Gradual fear generalization was also observed across all subjects in all ratings (all *p*s < 0.001) and in the FPS (*p* = 0.001) (Fig. S2), but not in the LPPs (i.e., GS+ > CS-, all *p*s > 0.1). Further details can be found in Supplement 2.

### Group differences in fear acquisition

*The means and standard deviations of the responses to the CS+ and the CS- in the acquisition phase by group are reported in* Table 2.

**Table 2:** Mean (M) and standard deviations (SD) by group for responses to the CS+ and the CS- for the acquisition and extinction phase.

	At-risk		Healthy controls	
	CS+	CS-	CS+	CS-
	*M(SD)*	*M(SD)*	*M(SD)*	*M(SD)*
*Acquisition*				
Expectancy	88.09 (14.31)	17.36 (22.53)	87.86 (12)	11.41 (15.53)
Valence	72.89 (18.12)	30.82 (27.37)	77.73 (19.69)	20.86 (20.32)
Arousal	72.89 (21.98)	33.84 (27.17)	76.00 (22.63)	26.27 (21.72)
Fear	64.11 (25.72)	30.11 (26.33)	58.91 (30.53)	14.36 (17.6)
Early LPP^a^	1.46 (3.67)	0.42 (4.46)	1.05 (3.14)	−0.10 (4.92)
Late LPP^a^	1.29 (6.59)	0.56 (7.13)	1.04 (4.46)	2.04 (12.12)
FPS^b^	56.81 (6.42)	56.09 (6.76)	57.57 (6.91)	54.86 (5.89)
*Extinction*				
Expectancy	41.00 (32.33)	18.02 (24.82)	31.14 (27.45)	9.86 (17.58)
Valence	41.34 (28.44)	24.57 (26.38)	28.955 (26.96)	14.95 (21.01)
Arousal	48.05 (30.97)	29.00 (29.02)	31.77 (27.36)	17.91 (24.39)
Fear	40.34 (29.65)	23.98 (25.74)	26.73 (26.81)	10.68 (18.28)
Early LPP^a^	1.16 (3.56)	2.31 (3.21)	1.59 (3.08)	2.48 (3.40)
Late LPP^a^	0.34 (4.19)	1.93 (4.25)	1.23 (4.77)	2.78 (5.37)
FPS^b^	48.29 (3.60)	47.07 (3.38)	47.35 (3.39)	47.50 (3.28)
*ExtinctionF*^*c*^				
Early LPP^a^	1.16 (3.55)	2.31 (3.21)	1.59 (3.08)	2.48 (3.40)
Late LPP^a^	0.34 (4.19)	1.93 (4.25)	1.23 (4.77)	2.78 (5.37)
*ExtinctionL*^*d*^				
Early LPP^a^	1.74 (3.90)	1.45 (3.50)	2.36 (4.41)	1.87 (4.42)
Late LPP^a^	1.95 (4.00)	1.67 (5.21)	1.84 (4.29)	2.04 (5.44)

^a^Late positive potential (LPP) values are represented in amplitudes (averaged across the electrode cluster) and are calculated over trials during the second block of acquisition (i.e., last 10 trials), first block of extinction (i.e., first 10 trials) and the last block of extinction (i.e., last 10 trials).

^b^Fear potentiated startle (FPS) magnitudes are in t-scores and ratings in values ranging from 0 to 100.

^c^*ExtinctionF*: The first block of extinction (i.e., the first 10 CS trials).

^d^*ExtinctionL*: The last block of extinction (i.e., the last 10 CS trials).

**Fig. 2:**
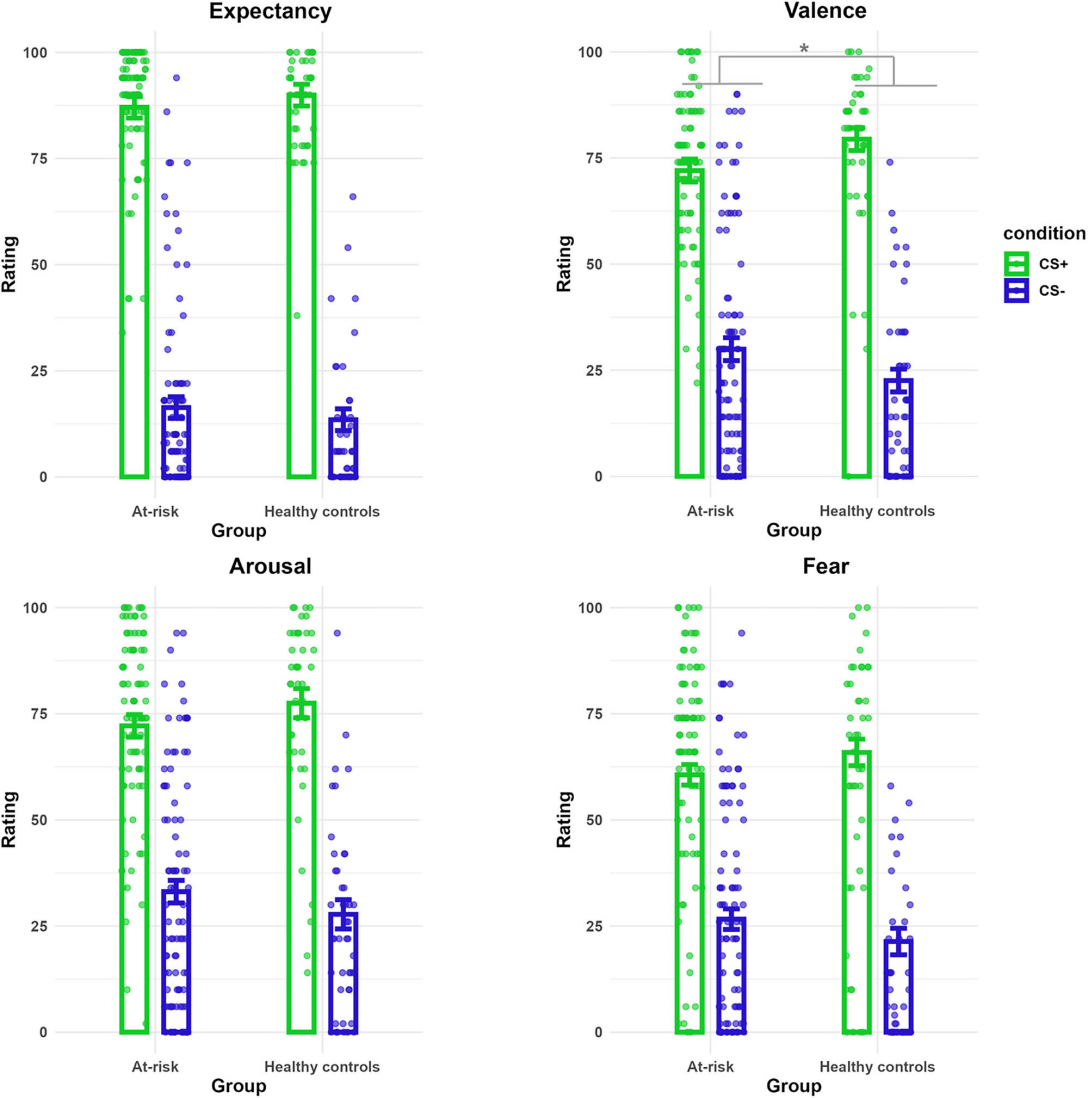
Subjective ratings given for CS+ and CS- following the acquisition phase per group. Bar plots display the mean expectancy, valence, arousal and fear ratings given to each CS type after the acquisition phase, separately for each group. Individual data points are shown for each participant. Error bars represent the standard error of the mean. *p < 0.05.

A significant group x CS type interaction was found for valence ratings, indicating a reduced CS+ vs. CS− discrimination in the at-risk group (*p* = .011). No significant interactions were found for expectancy, fear, or arousal ratings (See Fig. 2 and Table 3). No group differences were found in either early or late LPPs, across all trials or when analyzing only the second block (all *p*s > 0.25). Also, no significant group differences were found for the FPS (*p* = 0.29).

**Table 3:** Fixed effects estimated in the linear mixed-effect models for the acquisition and extinction ratings.

	Expectancy		Valence		Arousal		Fear	
	*b(SE)*	*CI*	*b(SE)*	*CI*	*b(SE)*	*CI*	*b(SE)*	*CI*
*Acquisition*								
(Intercept)	11.41 (2.62)**	6.25 to 16.57	20.86 (3.35) **	14.27 to 27.45	26.27 (3.60)**	19.18 to 33.37	14.36 (3.87)**	6.75 to 21.98
CS type	76.45 (3.71)**	69.16 to 83.75	56.86 (4.73) **	47.54 to 66.18	49.73 (5.10)**	39.69 to 59.76	44.55 (4.71)**	35.26 to 53.83
group	5.95 (3.21)	−03.6 to 12.27	9.95 (4.10)*	1.88 to 18.03	7.57 (4.41)	−1.12 to 16.26	15.75 (4.74)*	6.42 to 25.08
CS type x group	-5.73 (4.54)	−14.66 to 3.21	−14.80 (5.80)*	**−26.21** **to** **3.38**	−10.68 (6.24)	−22.97 to 1.61	−10.55 (5.77)	−21.91 to 0.82
*Extinction*								
(Intercept)	7.95 (2.67)*	2.71 to 13.20	15.86 (3.62)**	8.75 to 22.98	16.14 (3.54)**	9.18 to 23.09	10.64 (3.42)*	3.92 to 17.36
CS type	83.09 (3.89)**	75.45 to 90.73	61.00 (5.04)**	51.09 to 70.91	56.00 (5.03)**	46.62 to 66.38	46.05 (4.75)**	36.72 to 55.37
group	3.80 (3.27)	−2.62 to 10.27	12.20 (4.43)*	3.49 (20.92)	12.00 (4.34)*	3.48 to 20.52	12.91 (4.19)*	4.68-21.14
phase	1.91 (4.20)	−6.34 to 10.16	−0.91 (4.21)	−9.18 to 7.36	1.77 (4.64)	−7.33 to 10.88	0.05 (4.10)	−8.01 to 8.10
CS type x group	−3.80 (4.76)	−13.16 to 5.56	−12.05 (6.18)	−24.18 to 0.09	−10.09 (6.16)	−22.19 to 2.01	−1.30 (5.81)	−12.71 to 10.12
CS type x phase	−61.82 (4.67)**	−71.00 to 52.64	−47.00 (5.68)**	−58.15 to 35.85	−42.64 (5.84)**	−54.11 to 31.17	−30.00 (5.26)**	−40.33 to 19.67
group x phase	4.36 (5.14)	−5.74 to 14.47	−2.59 (5.15)	−12.72 to 7.53	−0.91 (5.68)	−12.06 to 10.24	0.39 (5.02)	−9.48 to 10.26
CS type x group x phase	5.50 (5.72)	−5.74 to 16.74	14.82 (6.95)*	**1.16** **to 28.48**	15.27 (7.15)*	**1.22** **to 29.32**	1.61 (6.44)	−11.04 to 14.27

*Note*. For the intercept and each fixed effect, 95% confidence intervals (*CI*) are presented alongside unstandardized beta coefficients (*b*) and the standard error (*SE*). Reference level for the factor CS type is set to CS-, for the factor group to healthy controls, and for the factor phase to Generalization, thus representing the *CS+ vs. CS-* discrimination*, At-risk vs. Healthy controls* and *Extinction vs. Generalization*, respectively. Bold texts represent the significant results supporting the hypotheses (i.e., group differences in acquisition and extinction learning).

*p < 0.05, **p < 0.01.

### Group differences in fear extinction

**Fig. 3:**
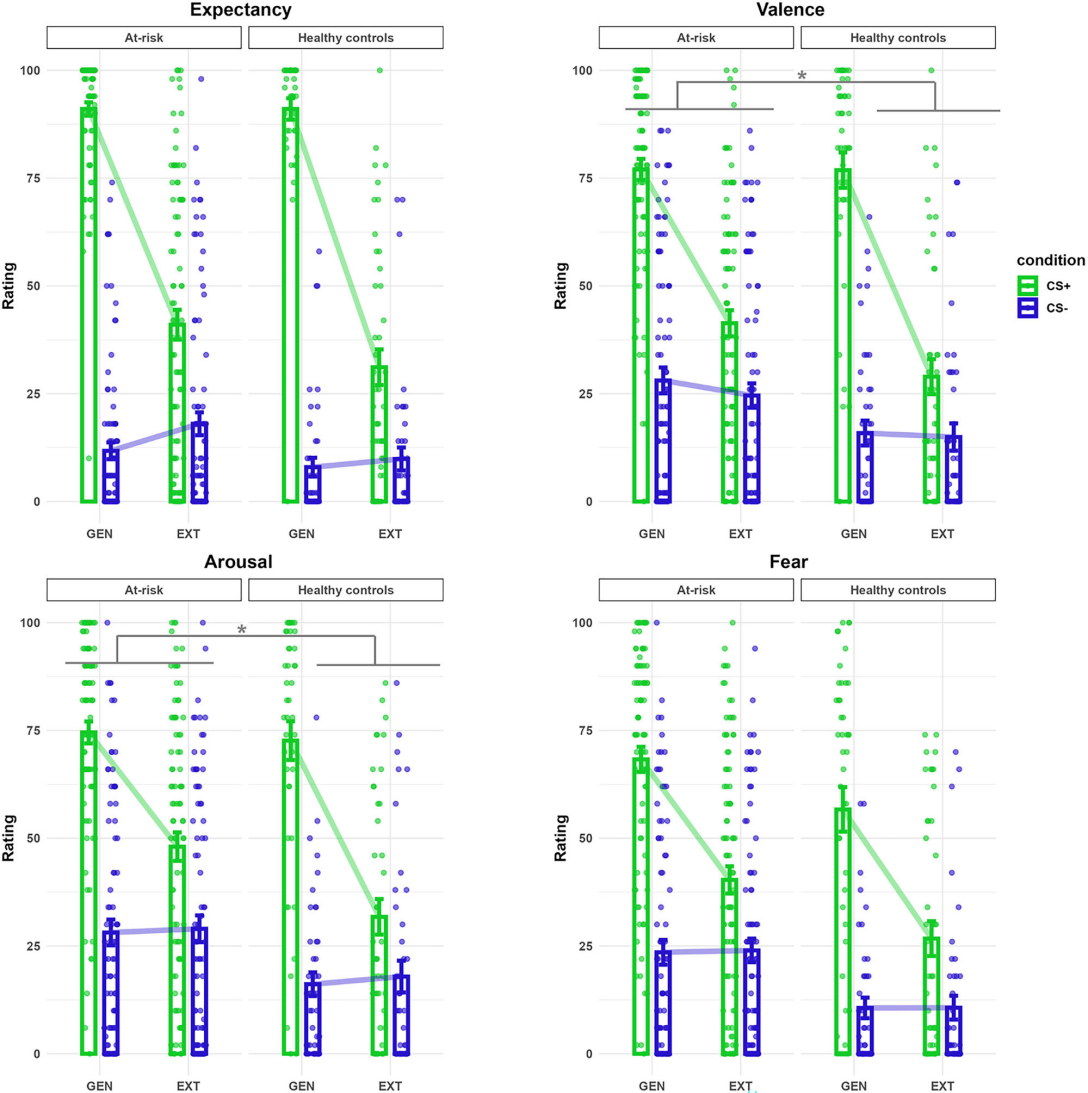
Subjective ratings given for each CS following the generalization and the extinction phase per each group. Bar plots display the mean expectancy, valence, arousal and fear ratings given to each CS type after the generalization (GEN) and after the extinction (EXT) phase (CS+ is represented in green, CS- in blue) separately for each group. Individual data points are shown for each participant. Error bars represent the standard error of the mean. *p < 0.05.

Valence and arousal ratings showed significant group × CS type × phase interactions. Compared to HCs, the at-risk group exhibited slower extinction, as reflected in a smaller decrease in the CS+ vs. CS− discrimination from generalization to extinction (all *p*s < 0.04) (see Fig. 3 and Tables 2 and 3). For both valence and arousal, CS+ ratings decreased from generalization to extinction in both groups, CS− ratings remained stable, and only the at-risk group still showed a significant CS+ versus CS− difference after extinction. Expectancy and fear ratings did not differ significantly between groups (all *p*s > 0.33). No significant group differences were found during extinction in LPPs or FPSs (all *p*s > 0.49).

See Supplement 3 for complete test statistics and follow-up analyses.

### Exploratory analyses

#### Generalization

The at-risk group demonstrated overgeneralization in the expectancy ratings (see Fig. S3 and Table S1) as reflected by smaller linear deviation scores (*p* = 0.02) (Supplement 4).

#### Dimensional analysis with CAPE

There was a significant CS type x CAPE scores interaction in expectancy (*p* = 0.009), valence (*p* = 0.021), and arousal (*p* = 0.006) ratings during acquisition (Fig. S4). This indicates decreasing CS discrimination with increasing CAPE scores. We also found a negative association between CAPE scores and the linear deviation scores of expectancy ratings (*p* = 0.010) (Fig. S5) (Supplement 5).

#### Subgroup analysis comparing PP, CHR and HC

Subgroup differences only emerged between PP and HC: As revealed by the group x CS type interaction, the PP group showed smaller CS discrimination during acquisition in valence (*p* = 0.005) and fear ratings (*p* = 0.041). The PP group also showed reduced CS discrimination during acquisition as compared to the CHR group in FPS (*p* = 0.011). Lastly, the PP group also showed overgeneralization in expectancy ratings (*p* = 0.009), as well as slower extinction in both valence (*p* = 0.016), and arousal ratings (*p* = 0.022) (Supplement 6).

#### Re-analysis including gender, education and age as covariates

The group x CS type interaction remained significant and unchanged in both effect size and significance for valence ratings during acquisition, and expectancy ratings during generalization. The group x CS type x phase interaction also remained significant for the valence and arousal ratings during extinction phase. This indicates that demographic variables did not account for the variance underlying these effects, suggesting that the observed group differences are robust and independent of the identified gender and education-related differences (Supplements 7 and 8).

#### Correcting for multiple comparisons

After applying the Bonferroni correction for multiple comparisons across the seven outcome variables (four self-reported ratings, two LPP components, and the startle response), several findings no longer met the criteria for statistical significance. These included the valence rating difference between at-risk and HCs during acquisition, and the valence and arousal ratings differences between groups during extinction. We applied the Bonferroni correction to identify which effects can be considered statistically robust. All manipulation-check findings reported in the “Results” section survived this correction.

## Discussion

Here, we investigated fear acquisition and extinction learning in individuals at risk of developing psychosis using a well validated paradigm. Although minor procedural adjustments were required for EEG compatibility, the paradigm we implemented adheres to the established standards of differential fear conditioning research^14^. Following the acquisition phase, the at-risk group showed a reduced discrimination in their self-reported affective response to the threat versus the safe CS compared to HCs. The same pattern was also demonstrated in the dimensional analysis conducted with the CAPE score (i.e., reduced discrimination with increasing CAPE scores). Furthermore, this discrimination showed a slower extinction in the at-risk group. These effects were observed only in valence and arousal ratings, not in neural (LPP) or physiological (FPS) measures.

The reduced CS+ vs. CS− discrimination of the at-risk group in valence ratings during acquisition partially supported our hypothesis and aligns with previous research demonstrating reduced differential responding in subjective ratings of affect in psychosis^23^. Follow-up pairwise comparisons of CS+ and CS- ratings between groups did not reach statistical significance, which suggests that differences in both CS+ and CS- responses jointly contributed to the attenuated discrimination in the at-risk group. Taken together with the absence of an effect on the autonomic affective FPS and LPP, these results tentatively suggest that individuals at risk of psychosis show aberrant fear acquisition primarily at the level of conscious affective responses. Dissociation between self-reported and physiological outcomes accords with dual-process models of fear learning. These models posit that affective and cognitive ratings reflect a distinct process from physiological responses, and they have been used to explain differences in outcomes in fear conditioning research^50,51^. Interestingly, several other studies also found differences in fear learning between clinical samples and healthy controls to emerge in the subjective ratings but not in the autonomic or neural outcomes^52,53^. The same pattern has been found in related areas, such as in experimental research on emotion regulation^54^ or stress-responses and recovery^55,56^, and may be driven by a stronger divergence between subjective feelings and evaluations on the one hand and biological and neural process on the other hand in people with mental disorders compared to healthy controls. In accord with this, it was demonstrated that both psychosis and depression are characterized by mismatches between autonomic stress indicators (i.e., heart rate, SCRs) and self-reported stress which was associated with low emotional awareness^57^.

These findings are also partly consistent with prior research showing that individuals with schizophrenia and those at risk for psychosis display reduced differentiation in their affective responses, e.g., perceiving pleasant stimuli as less positive, and neutral stimuli as more negative^58^. Our results extend this literature by demonstrating that individuals at risk for psychosis also show impaired discrimination between learned threat and safety cues, as reflected in their affective ratings. Having medication inference ruled out as we employed people with an at-risk state who are not on antipsychotic medication, we can claim that this lack of differential subjective affective responding to threat cues could be more directly related to, and explain, the core features of psychotic experiences. In this sense, this pattern may reflect a broader disruption in predictive coding^59^, in which prior expectations of threat fail to regulate affective responses appropriately, leading to heightened affective uncertainty and possibly contributing to delusions and accompanying the emotional distress. This may also be viewed through the lens of aberrant salience theory^13^, which associates psychotic symptoms with the assignment of aberrant significance to otherwise neutral events.

The lack of significant group differences in expectancy ratings suggests that individuals at risk of developing psychosis show a healthy pattern of conscious cognitive evaluations of contingencies and learn the task correctly. Again, this fits in with the results of fear conditioning studies in anxiety disorders, with a recent meta-analysis indicating no differences to HCs in the differential expectancy ratings^3^. To our knowledge, no fear-conditioning study on psychosis has included expectancy ratings. However, the lack of group-differences in the LPPs does not align with prior findings of reduced differential P300 amplitudes in individuals with a CHR status^27^. In that study, an at-risk sample consisting exclusively of CHR was tested, whereas our sample included a broader spectrum of at-risk severity. However, our exploratory subgroup analysis did not indicate that LPP aberrancies during acquisition were more pronounced in the CHR subsample either (see Supplement 6). The use of social stimuli in related research (i.e., neutral faces) may have accounted for the differences in findings as CHR individuals have been found to show increased neural activation to neutral facial expressions^60^.

In regard to extinction, we found the same dissociation between self-reported and physiological outcomes, with deviations from HCs only showing up in the self-report measures. The at-risk group exhibited a slower reduction in differential CS responses in valence and arousal ratings which suggests an impaired ability to update the affective responses to a CS in response to new information. This is even more noteworthy given that the at-risk group exhibited a significantly reduced discrimination between CS+ and CS− in valence ratings compared to the healthy controls at the end of the acquisition and generalization phase. This seems to indicate a particular resistance to extinguishing subjective valence-related responses in the individuals at risk. This aligns with prior research demonstrating impaired extinction in individuals with high delusion-proneness^29^ and could be used as a starting point to further elucidate the exact mechanisms of extinction learning in people with persistent delusions^61^.

The high prevalence of affective and anxiety disorders within our at-risk sample raises the question of whether the observed fear acquisition and extinction learning alterations are specific to psychosis risk or reflect broader emotional dysregulation that is prevalent across various diagnoses. However, this comorbidity mirrors the clinical profiles of individuals at risk for psychosis^62^, suggesting that our findings have ecological validity for this population. Also, the finding aligns with studies showing altered extinction learning in people with a full diagnosis of psychosis^21^. Overall, the failure to extinguish learned threat associations points to a rigidity in conscious affective processing in individuals at risk for psychosis. This might contribute to the persistence of maladaptive emotional responses that is also evident in other mental disorders, such as anxiety^3^, and can be considered a transdiagnostic feature.

### Limitations

The small CHR subgroup limits subgroup analysis reliability. Also, the intensive outreach to the general public may have diluted the pre-test risk^63^. On the other hand, the overall comorbidity rates in our combined at-risk sample are comparable with those in large scale studies using established at-risk criteria^62^. This indicates that the risk profile in our sample corresponds to the established profiles. However, the sample was slightly skewed towards women with higher education levels, although these demographics did not significantly impact the primary outcomes.

Additionally, the use of retrospective ratings (i.e., ratings collected after phases) restricted trial-level analysis. The EEG-optimized timing in the design did not support reliable SCR analysis^64^, therefore, as pre-registered, we did not include SCR data in our hypothesis testing. Moreover, for hypothesis testing, multiple comparisons were not corrected across outcome modalities as each measure reflects a distinct aspect of fear learning and thus provides complementary information^14^. While this approach aligns with current practice in the field, we acknowledge that it increases the risk of Type I errors. To estimate the potential impact of multiple testing, we additionally applied a conservative Bonferroni correction in an exploratory analysis. Under this stricter criterion, some effects no longer reached significance. This suggests that group differences are less pronounced or absent when controlling for family-wise error. Nevertheless, the broader pattern remains in line with existing literature.

## Conclusion

We found people with psychotic vulnerability to show impaired fear learning with less differentiation between fear-associated and neutral stimuli and with a heightened resistance to fear extinction, particularly in learned evaluations of valence and arousal. This suggests heightened persistence of learned affective responses when threat-related cues no longer signal danger which potentially contributes to difficulties in adapting established emotional responses to changing environmental contingencies. In light of these findings, we propose that learned associations of valence and arousal of fear and safety associated stimuli may represent an early – albeit possibly non-specific - marker of psychotic vulnerability. Our findings may help refine therapeutic strategies by targeted interventions focused on recalibrating emotional learning related to threat-associated cues.

## Supplementary information

Supplements

## Data Availability

The datasets generated during and/or analyzed during the current study are available in the OSF repository (https://osf.io/fj9cg/).

## Code availability

Analysis code(s) used in the processing of datasets are available in the OSF repository (https://osf.io/fj9cg/). The software(s) as well as their versions used are reported in the following sections.
