# Supplementary material for: The role of fear learning in the development of psychosis: an EEG study utilizing a differential fear conditioning paradigm in people with psychotic vulnerability
Source: Schizophrenia (Heidelb). 2026 May 15;12(1):45. doi: 10.1038/s41537-026-00761-y (PMC13179330; doi:10.1038/s41537-026-00761-y)
Supplement: Supplementary file 1 — Supplements [file 41537_2026_761_MOESM1_ESM.docx]

**SUPPLEMENTARY INFORMATION**

**The role of fear learning in the development of psychosis: An EEG study utilizing a differential fear conditioning paradigm in people with psychotic vulnerability.**

Özyagcilar *et al.*

**SUPPLEMENT 1**

**Neurophysiological Data Acquisition and Processing – Additional details**

**Electroencephalogram (EEG)**

In case any channels were interpolated prior to the Independent Component Analysis (ICA), the rank/number of components was reduced by the number of channels interpolated. ICA decomposition was also preceded by an additional 1 Hz high-pass filter and rejection of high-artifact segments (i.e. 1s epochs, >2 SD joint probability) (Viola et al., 2010; Winkler et al., 2015). After manually selecting the components representing artefacts, the ICA decomposition and weights were then added to the original data set (which is continuous and not filtered with the 1 Hz high-pass filter). The detected bad components were then removed from the data.

Minimum 50% of all of the trials (Luck, 2014) are ensured for the subsequent event-related-potential (ERP) analysis with regards to each phase (or each block during Acquisition and Extinction).

**Fear Potentiated Startle (FPS)**

Two Ag/AgCl electrodes had a sensor diameter of 6 mm. Before the electrode application, an abrasive peeling gel was applied onto the skin and later cleaned up with alcohol. The data was pre-processed in the Brain Vision Analyser, using filtering (28–500 Hz, 24 dB/octave roll-off), rectification, baseline correction (baseline = 50 ms prior to startle probe onset), and integration (9 ms moving average). For each stimulus in each phase, segments from -500 ms to 250 ms relative to the startle probe were extracted. This segmentation was followed by detecting segments with excessive noise or spontaneous blinks that were scored as "missings”. This detection took place automatically using the EMG Onset Search macro. In a time window (-50 ms to 20 ms relative to the startle probe, i.e., refractory period), deviations ≥ 4 SD from either of two baselines (-500 to -300 ms and -300 to -100 ms relative to the startle probe) were scored as missing and omitted from analyses and further detection of startle blinks. Two baselines were used to avoid spontaneous blinks in either baseline period that could result in the inability of the algorithm to pick up blinks in the refractory period. Using the same algorithm, startle probe onset with a time window from 20 to 120 ms after startle probe, was determined by deviations ≥ 4 SD from the baseline -50 to 0 ms relative to startle probe and the highest peak was scored in a 20 ms to 150 ms window relative to the startle probe. Undetectable reactions (i.e., segments that are neither “missings” nor contain > 4 SD baseline deviations in the response time window) were scored as zero and included in the analyses (i.e., startle magnitude, cf. Blumenthal et al., 2005). To ensure the availability of sufficient startle reactions, participants were excluded from the FPS analyses if they showed zeros or missings to > 66% of the startle probes, including the 10 startle trials, presented in the initial startle habituation phase[cf., (Kuhn et al., 2020)]. As a result, 25 participants were excluded from FPS analyses: Psychosis-proneness (PP); *n* = 13, clinical-high-risk (CHR); *n* = 6, healthy controls (HC); *n* = 6.

**Additional details on the paradigm**

The paradigm was presented using the Presentation Software (Version 23.0, Neurobehavioral Systems Inc). Two different orders of trials were used in a pseudorandomized manner. In one order the reinforced (i.e., followed by the electro-tactile stimulation) conditioned stimuli (CS+) appeared first during each phase, and in the other one the unreinforced conditioned stimuli (CS-).

**SUPPLEMENT 2**

**Manipulation Check**

**Acquisition**CS+ elicited significantly higher responses than CS- across all ratings. For expectancy, the fixed effect of CS type was significant, *b* = 72.64, *SE* = 2.15, *t*(260) = 33.86, *p* < .001. Similarly, significant effects were found for valence (*b* = 47.00, *SE* = 2.76, *t*(260) = 17.03, *p* < .001), arousal (*b* = 42.61, *SE* = 2.95, *t*(260) = 14.44, *p* < .001), and fear ratings (*b* = 37.52, *SE* = 2.75, *t*(260) = 13.66, *p* < .001).
No significant difference was found in early late positive potentials (LPPs) across all acquisition trials (*b* = 0.51, *SE* = 0.40, *t*(1577) = 1.29, *p* = .20). However, when restricting analysis to the second half of the trials, CS+ elicited significantly larger LPPs than CS- (*b* = 1.08, *SE* = 0.47, *t*(1577) = 2.28, *p* = .02). For late LPPs, no significant effects were found in either the full acquisition trials (*b* = –0.51, *SE* = 0.75, *t*(1577) = –0.68, *p* = .50) or the second half (*b* = 0.15, *SE* = 0.89, *t*(1577) = 0.17, *p* = .87).
There was no significant difference between the CSs in the FPS data during acquisition *b* = 1.43, *SE* = 0.89, *t*(210) = 1.61, *p* =.11.

**Generalization**

Manipulation check model of generalization included the fixed effect CS(/GS), which included five levels: CS+, GS+, GSU, GS-, CS-.
A gradual generalization pattern was observed in all ratings, with generalization extending to the GS+ (i.e., GS+ > CS-). A significant CS+ vs. CS- discrimination was observed for expectancy ratings (*b* = 80.56, *SE* = 2.42, *t*(653) = 33.27, *p* < .001) as well as for GS+ vs. CS- (*b* = 39.56, *SE* = 2.42, *t*(653) = 16.34, *p* < .001). Follow-up pairwise comparisons revealed that CS+ ratings were higher than those to all GSs (*ps* < .001), and GS +responses were higher than those to GSU (*t*(524) = 14.97, *p* < .0001) and GS- (*t*(524) = 16.46, *p* < .0001). For valence ratings, CS+ ratings were higher than CS- (*b* = 52.97, *SE* = 2.66, *t*(653) = 19.93, *p* < .001), as were GS+ ratings vs. CS- (*b* = 22.29, *SE* = 2.66, *t*(653) = 8.38, *p* < .001), and GS- was ratings were smaller than CS- (*b* = –5.30, *SE* = 2.66, *t*(653) = –1.99, *p* = .046). Pairwise comparisons confirmed CS+ > all GSs (*ps* < .001) and GS+ > GSU (*t*(524) = 9.66, *p* < .0001) and GS-(*t*(524) = 10.38, *p* < .0001). Similar patterns were found for arousal, with significant discrimination between CS+ vs. CS- (*b* = 49.77, *SE* = 2.63, *t*(653) = 18.93, *p* < .001) and GS+ vs. CS-(*b* = 22.15, *SE* = 2.63, *t*(653) = 8.42, *p* < .001), with follow-up comparisons showing CS+ > all GSs (*ps* < .001), GS+ > GSU (*t*(524) = 9.18, *p* < .0001) and GS- (*t*(524) = 9.64, *p* < .0001). Fear ratings followed the same pattern: CS+ vs. CS- (*b* = 45.18, *SE* = 2.45, *t*(653) = 18.41, *p* < .001), GS+ vs. CS- (*b* = 21.73, *SE* = 2.45, *t*(653) = 8.85, *p* < .001), and follow-up comparisons showing CS+ > all GSs (*ps* < .001), GS+ > GSU (*t*(524) = 9.40, *p* < .0001), GS+ > GS- (*t*(524) = 9.59, *p* < .0001).
For early LPPs, a significant CS+ vs. CS- discrimination was observed (*b* = 1.59, *SE* = 0.35, *t*(3938) = 4.52, *p* < .001). CS+ also elicited significantly higher amplitudes than GS+ (*z* = 2.98, *p* < .05), GSU (*z* = 5.47, *p* < .0001), and GS- (*z* = 4.28, *p* = .0002). GS+ vs. CS- discrimination was not significant, (*b* = 0.53, *SE* = 0.43, *t*(3938) = 1.58, *p =* 0.16. Late LPPs did not reveal any significant effects (*ps* > .1). Thus, we could not demonstrate the gradual fear generalization for the LPPs.

FPS responses were larger for CS+ than CS- (*b* = 6.82, *SE* = 0.85, *t*(515) = 8.05, *p* < .001) and for GS+ vs. CS- (*b* = 2.82, *SE* = 2.42, *t*(515) = 0.85, *p* = .001). Pairwise comparisons confirmed that CS+ elicited stronger responses than all GSs (*ps* < .001), and that GS+ > GSU (*t*(415) = 3.93, *p* = .001) and GS+ > GS- (*t*(414) = 3.49, *p* = .005).

**Extinction**All measures showed significant CS type × phase interactions, reflecting reduced CS+ vs. CS- differences from after generalization to after extinction.

The reduction was observed for expectancy, *b* = –58.15, *SE* = 2.69, *t*(517) = –21.60, *p* < .001. Also, CS+ ratings decreased significantly from generalization to extinction (*t*(131) = –29.15, *p* < .0001), whereas CS- remained stable (*t*(228) = 1.98, *p* = .20). The CS discrimination (i.e., CS+ > CS-) remained significant at extinction (*t*(243) = 10.01, *p* < .0001). For valence, the interaction was also significant (*b* = –37.12, *SE* = 3.29, *t*(517) = –11.27, *p* < .001), with CS+ decreasing (*t*(260) = –16.25, *p* < .0001), CS- unchanged (*t*(260) = –1.08, *p* = .70), and the CS discrimination remaining (*t*(232) = 5.44, *p* < .0001). Arousal ratings followed the same pattern (*b* = –32.45, *SE* = 3.42, *t*(517) = –9.50, *p* < .001), with CS+ decreasing (*t*(252) = –11.59, *p* < .0001), CS- stable (*t*(252) = 0.43, *p* = .97), and a significant CS discrimination at extinction (*t*(239) = 5.95, *p* < .0001). Fear ratings also showed a significant interaction (*b* = –28.92, *SE* = 3.03, *t*(517) = –9.56, *p* < .001), with CS+ decreasing (*t*(254) = –12.13, *p* < .0001), CS- unchanged (*t*(254) = 0.13, *p* = .99), and the discrimination remaining significant (*t*(228) = 5.96, *p* < .0001).
For early LPPs, the CS type × block interaction was significant (*b* = 1.42, *SE* = 0.26, *t*(3156) = 5.54, *p* < .001), indicating reduced differentiation from the first to last extinction block. The first block showed, however, an unexpected pattern of CS- > CS+ (*z* = 3.65, *p* = .001), which disappeared in the final block (*z* = 1.21, *p* = .062). No significant CS+ vs. CS- discrimination was found across all trials (*b* = 0.07, *SE* = 0.17, *t*(1577) = 0.40, *p* = .69). For late LPPs, the interaction was again significant (*b* = 1.70, *SE* = 0.37, *t*(3156) = 4.60, *p* < .001). CS+ responses increased from first to last block (*z* = 3.49, *p* = .002), early block showed CS- > CS+ (*z* = 3.65, *p* = .001), and no difference was observed in the final block (*z* = 0.28, *p* = .99). No overall CS+ vs. CS- discrimination was found across trials (*b* = –0.23, *SE* = 0.26, *t*(1577) = –0.89, *p* = .37).
The CS type × phase interaction was significant for FPS, *b* = –6.16, *SE* = –7.26, *t*(410) = 0.85, *p* < .001, reflecting a diminished CS+ vs. CS- difference in extinction compared to generalization. CS+ startle responses significantly decreased (*t*(195) = –10.07, *p* < .0001), while CS- responses remained unchanged (*t*(195) = –1.25, *p* = .60). The CS+ vs. CS- discrimination was no longer significant in extinction (*t*(195) = 1.05, *p* = .72).

**Figure S1.**

*Grand average ERP waveforms elicited by each CS/GS during each phase (across all participants)*


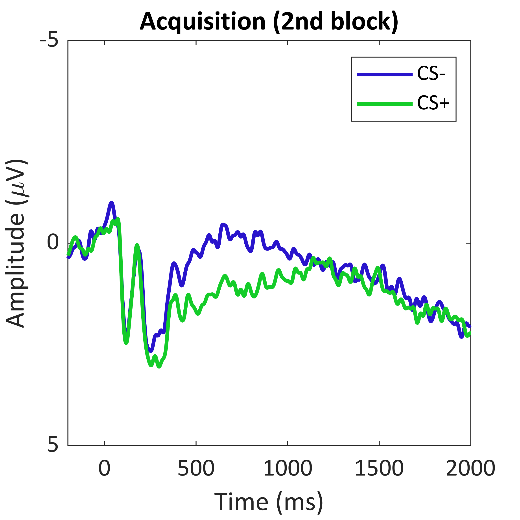

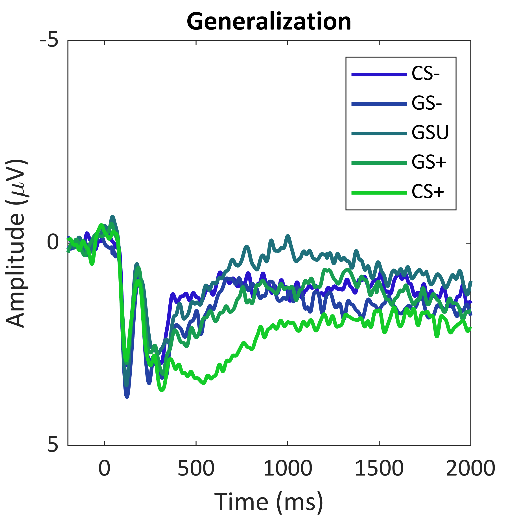

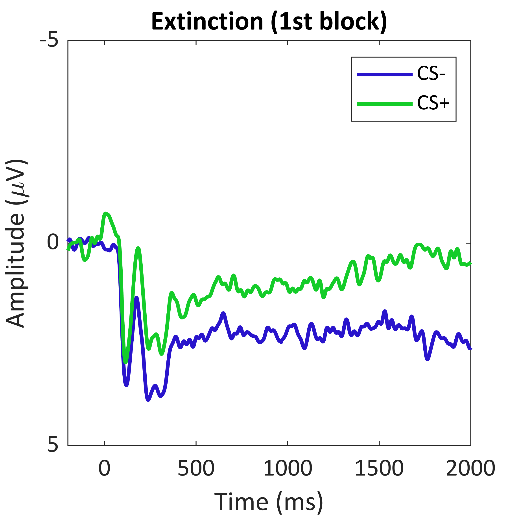

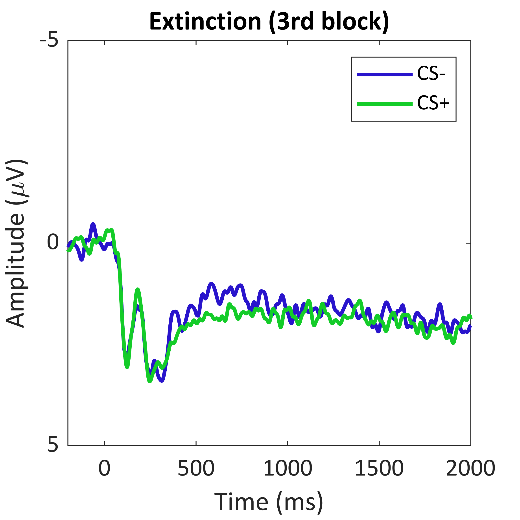

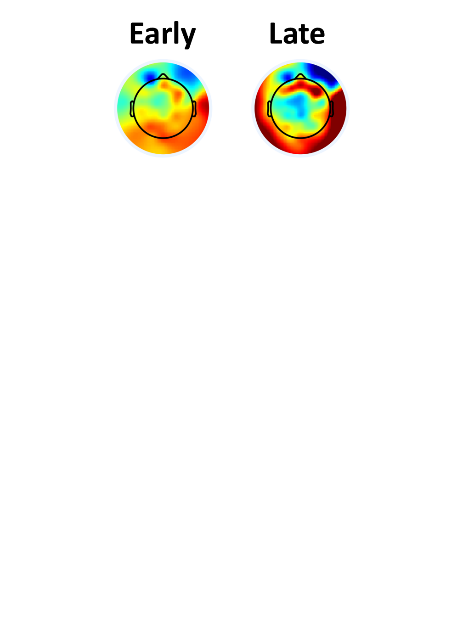

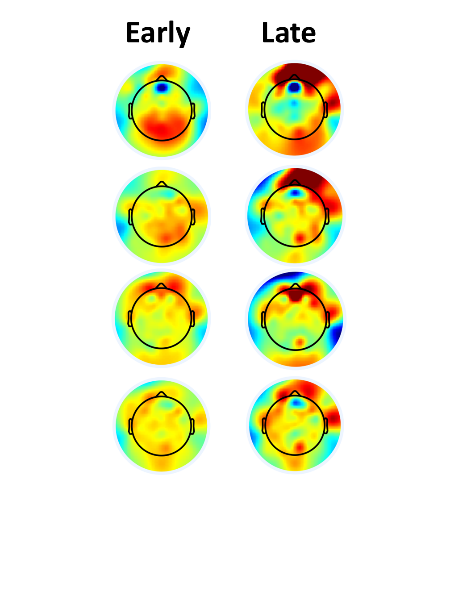

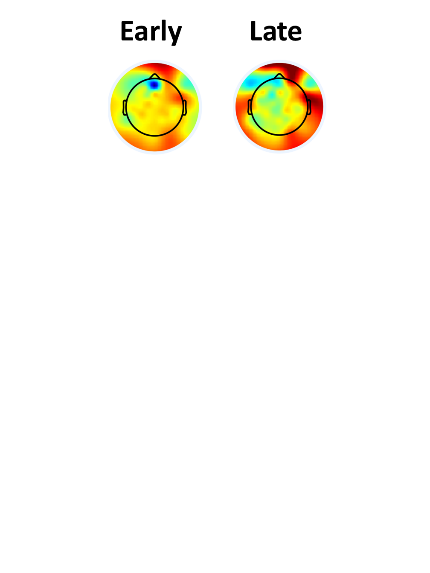

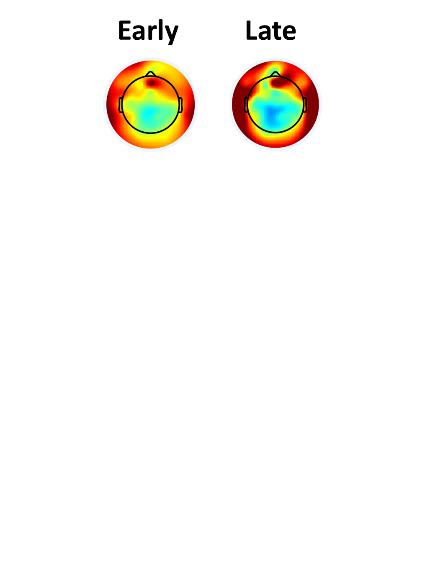

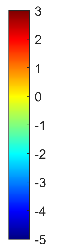


*Note.* Grand average event-related-potentials (ERP) waveforms are pooled across the electrodes PO1, PO2, Pz, O1, O2 and Oz, displaying the early and late time-window of the late positive potentials (LPPs). Difference scalp topographies are presented displaying the CS+ activity vs. the CS- (for generalization, from top to bottom: CS+, GS+, GSU and GS- vs. CS-) over the scalp.

**Figure S2.**

*FPS elicited during each phase (across all participants)*


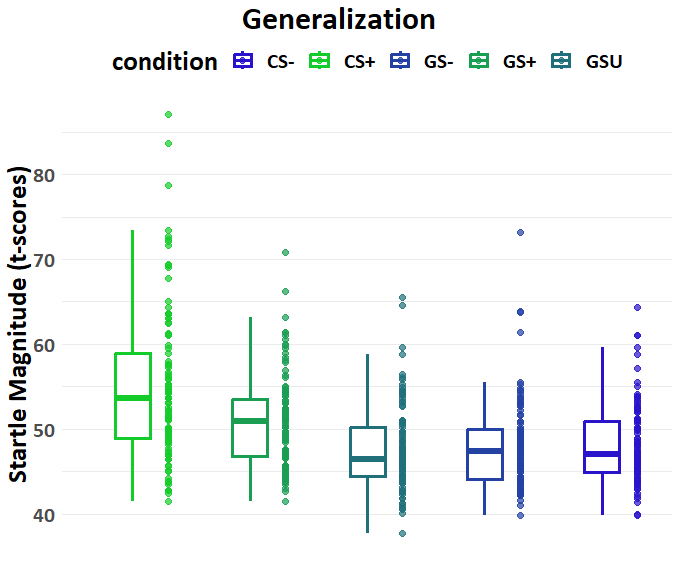

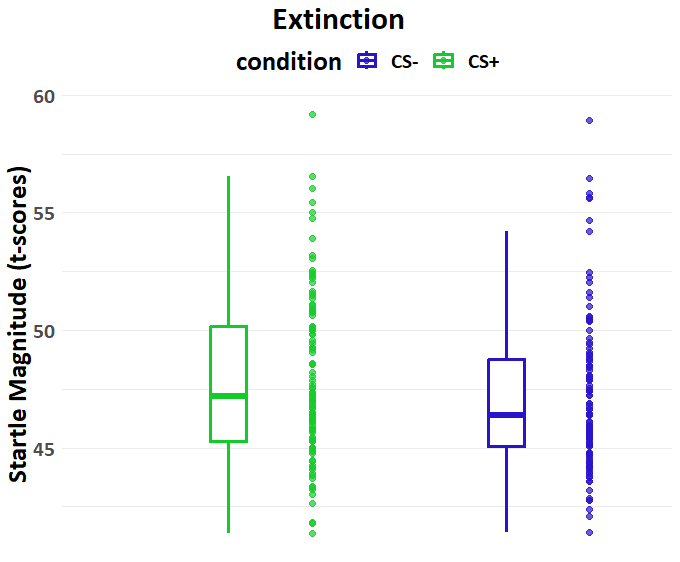

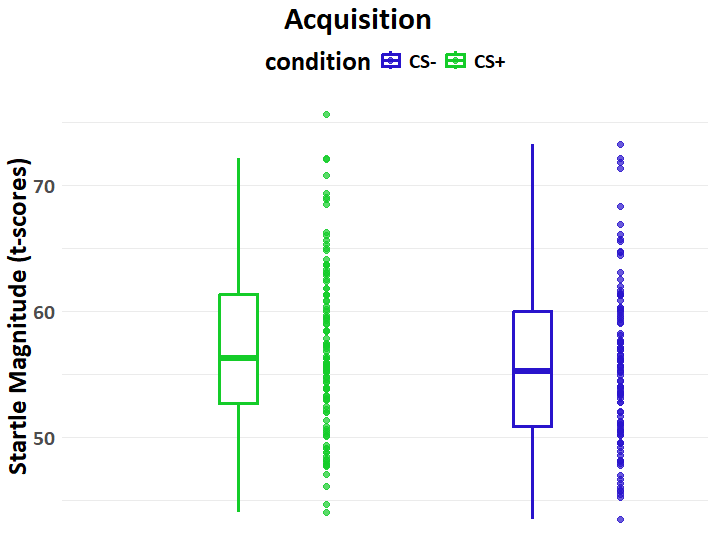


*Note.* Box plots display the calculated t-scores for the fear potentiated startle (FPS) magnitude per each phase and stimulus, across all participants. Next to the box plots, individual data points are shown for each variable.

**SUPPLEMENT 3**

**Group differences in acquisition and extinction (detailed results)**

**Acquisition**

The acquisition model revealed a significant interaction of group x CS type indicating smaller CS+/CS- discrimination of valence (*b* = -14.80, *t*(258) = -2.55, *SE* = 5.80, *p =* .011) in the at-risk group. Follow-up pairwise comparisons did not reveal any statistically significant difference between the two groups for the valence ratings given for the CS-, *t*(260) = -2.43, *p* = 0.07, or for the CS+, *t*(260) = 1.18, *p* = .64. No significant interactions were found for the expectancy, fear and arousal ratings (all *p*s > .06).

No significant group x CS type interaction was present neither for the Early (All trials: *b* = -0.23, *t*(1575) = -0.27, *SE* = 0.84, *p =* .79 / 2^nd^ block: *b* = -0.11, *t*(1575) = -0.11, *SE* = 1.01, *p =* .91) and nor for the Late LPPs (All trials: *b* = 1.80, *t*(1575) = 1.13, *SE* = 1.59, *p =* .26 / 2^nd^ block: *b* = 1.74, *t*(1575) = 0.92, *SE* = 1.89, *p =* .36) in the acquisition model, indicating similar levels of acquisition learning between the two groups at a neural level.

Similarly, no significant group x CS type interaction was found for FPS (*b* = -1.99, *t*(208) = -1.07, *SE* = 1.87, *p =* .29) in the acquisition model, indicating similar levels of acquisition learning between the two groups on a physiological level.

**Extinction**

The extinction model revealed a significant group x CS type x phase interaction for the valence ratings, *b* = 14.82, *t*(513) = 2.13, *SE* = 6.95, *p =* .03. From generalization to extinction, the absolute difference between CS+ and CS- decreased for HC (from 61.0 in generalization to 14.0 in extinction). This decrease was smaller for the at-risk group, (from 49.0 in generalization to 16.8 in extinction) suggesting that the at-risk group demonstrated a slower extinction. Follow-up pairwise comparisons revealed that in both groups, CS- ratings did not significantly differ between extinction and generalization (HC: *t*(258) = -0.22, *p* = 1.00; at-risk: *t*(258) = -1.18, *p* = .94), whereas CS+ ratings were significantly lower after extinction compared to generalization (HC: *t*(258) = -11.39, *p* < .0001; at-risk: *t*(258) = -11.99, *p* < .0001). After the extinction phase, the CS+ vs CS- difference was still significant for the at-risk group, *t*(229) = 4.70, *p* < .0001; however not for HC, *t*(229) = 2.78,  *p* = .11.

The same pattern of results were observed for the arousal ratings: A significant group x CS type x phase interaction was also revealed for the arousal ratings, *b* = 15.27, *t*(513) = 2.14, *SE* = 7.15, *p =* .03. From generalization to extinction, HC showed a larger decrease in the absolute difference between CS+ and CS- (from 56.50 in generalization to 13.86 in extinction), as compared to the at-risk group (from 46.41 in generalization to 19.05 in extinction), suggesting that the at-risk group demonstrated a slower extinction. Follow-up pairwise comparisons showed that CS- ratings did not significantly differ between extinction and generalization in either group (HC: *t*(249) = 0.38, *p* = .99; at-risk: *t*(249) = 0.26, *p* = 1.00), while CS+ ratings were significantly lower after extinction compared to generalization (HC: *t*(249) = -8.82, *p* < .0001; at-risk: *t*(249) = -8.09, *p* < .0001). Furthermore, after the extinction phase, the CS+ vs CS- difference was still significant for at-risk group, *t*(235) = 5.36, *p* < .0001; however not for HC, *t*(235) = 2.76, *p* = .11.

Interactions for expectancy and fear ratings did not reach significance (all *p*s > .33).

The extinction model did not reveal a significant group x CS type x block interaction neither for the Early LPPs (*b* = 0.08, *t*(3152) = 0.14, *SE* = 0.54, *p =* .89), nor for the Late LPPs (*b* = 0.53, *t*(3152) = 0.67, *SE* = 0.79, *p =* .50).

Similarly, in the extinction model, no significant group x CS type x block interaction was found, *b* = 0.55, *t*(406) = 0.31, *SE* = 1.78, *p =* .76.

**SUPPLEMENT 4**

**Group differences in fear generalization learning**

The generalization model (testing the group differences) included the interaction term CS(/GS) type (which, in addition to CS+, CS-, also included the GS levels: GS+, GSU, GS-) x group (At-risk vs. HC). In order to compare the degree of generalization between groups, the linear deviation score (LDS) was also calculated using the following formula: [(CS+ + CS−)/2 – (GS+ + GSU + GS-)/3] (Stegmann et al., 2019), whereby lower scores indicate more linear generalization gradient and therefore pinpoint to overgeneralization. This score was used as the DV in an independent samples t-test with group as the between-subjects variable.

In the generalization model, the only significant group x CS(/GS) type interaction appeared in the expectancy rating and indicated a larger GS+ vs. CS- discrimination in the at-risk group compared to the HCs (*b* = 14.41, *t*(648) = 2.83, *SE* = 5.09, *p =* .005). Follow-up pairwise comparison indicated no significant differences for responses to CS- (*t*(577) = -0.96, *p* = 0.99), but revealed that higher expectancy responses were given to the GS+ in the at-risk group vs. the HCs, (*t*(524) = 4.59, *p* = .0002) (see Figure S3 and Table S1).

We found a significant group difference in LDS only for the expectancy ratings (*t*(130) = -2.46, *p* = .02); indicating smaller LDS and therefore a more linear generalization gradient in the at-risk group vs. the HCs. The LDS of the other ratings did not indicate significant differences (valence: *p* = .08, arousal: *p* = .4, fear: *p* = .88), neither did the interaction terms (with the GS+) within the generalization model in other ratings (valence: *p* = .52, arousal: *p* = .6, fear: *p* = .23). The only exception was the valence ratings in the generalization model, whereby a group x CS/GS type interaction emerged at the CS+ level (i.e., CS+ vs. CS-, *b* = -12.05, *t*(648) = 2.15, *SE* = 5.61, *p =* .032); indicating smaller discrimination in the valence ratings between the CS+ and the CS- in the at-risk group vs. the HCs.

For Early LPPs, no significant interaction was found in the generalization model (all *p*s > .6). No statistically significant difference for the LDS of the Early LPPs (averaged across the cluster) between the groups was found either, (*t*(130) = -0.03, *p* = .97). Similarly, for Late LPPs (averaged across the cluster), no significant interaction was emerged for any of the stimuli (all *p*s > .3). Also, the LDS between the groups did not significantly differ either, (*t*(130) = -0.45, *p* = .66). Similarly, in the generalization model, no significant interaction was found (all ps > 0.4) for the FPS. The comparison of the LDS of FPS did not reveal a significant group difference either, (*t*(104) = 0.51, *p* = .61).

**Figure S3.**

*
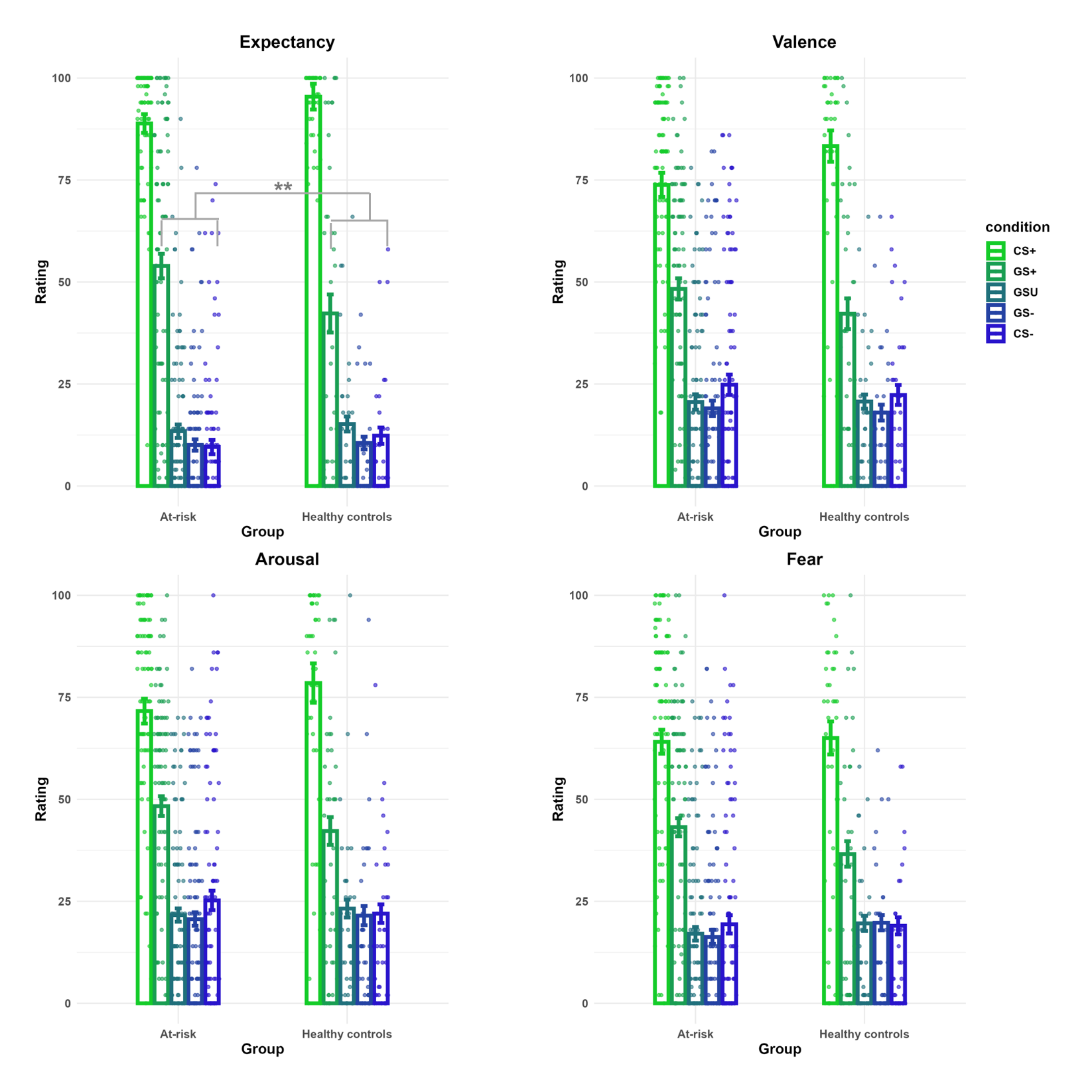
Subjective ratings given for each CS/GS following the generalization phase per each group*

*Note.* Bar plots display the mean expectancy, valence, arousal and fear ratings given to each CS/GS type after the generalization phase, separately for each group. Individual data points are shown for each participant. Error bars represent the standard error of the mean. * *p* < .05 ** *p* < .01 *** *p* < .001

**

**Table S1.**

*Mean (M) and standard deviations (SD) of the variables for each CS/GS type and group (during generalization)*

| **Condition** | **Expectancy M (SD)** | **Fear M (SD)** | **FPS** ^a^ **M (SD)** | **Valence M (SD)** | **Arousal M (SD)** | **Early LPP** ^b^ **M (SD)** | **Late LPP** ^b^ **M (SD)** |
| --- | --- | --- | --- | --- | --- | --- | --- |
| **Group** | **At-risk / HC ^c^** | **At-risk / HC** | **At-risk / HC** | **At-risk / HC** | **At-risk / HC** | **At-risk / HC** | **At-risk / HC** |
| CS+ | 91.05 (14.57) / 91.05 (16.48) | 68.30 (27.34) / 56.68 (34.20) | 55.27 (9.57) / 54.44 (8.06) | 77.02 (22.88) / 76.86 (27.18) | 74.55 (23.88) / 72.64 (29.78) | 2.74 (3.30) / 2.97 (2.76) | 1.45 (4.28) / 2.88 (4.40) |
| GS+ | 56.11 (33.49) / 37.91 (37.50) | 47.34 (27.32) / 28.23 (29.27) | 51.54 (5.80) / 50.00 (4.76) | 51.55 (26.93) / 35.77 (31.14) | 51.25 (25.28) / 36.36 (28.54) | 1.82 (3.22) / 1.63 (3.84) | 1.01 (3.42) / 1.54 (6.82) |
| GSU | 15.64 (19.67) / 10.82 (16.40) | 21.23 (21.61) / 11.23 (15.87) | 47.36 (4.29) / 48.19 (6.08) | 23.82 (22.62) / 14.23 (16.63) | 24.55 (21.60) / 17.36 (19.67) | 0.77 (3.19) / 1.37 (3.19) | 0.12 (3.94) / 1.61 (5.31) |
| GS- | 12.23 (16.45) / 6.14  (10.70) | 20.45 (22.78) / 11.41 (16.28) | 47.64 (3.87) / 48.76 (6.92) | 22.27 (25.23) / 11.55 (15.22) | 23.57 (22.70) / 15.64 (19.64) | 1.56 (3.21) / 1.66 (2.86) | 1.58 (4.27) / 1.45 (4.17) |
| CS- | 11.75 (18.26) / 7.95  (14.43) | 23.55 (27.01) / 10.64 (15.78) | 48.20 (4.79) / 48.08 (4.61) | 28.07 (28.21) / 15.86 (19.11) | 28.14 (28.00) / 16.11 (8.15) | 1.18 (3.16) / 1.32 (3.80) | 0.97 (5.34) / 1.47 (5.20) |

^a^ Fear potentiated startle (FPS) magnitudes are in t-scores and ratings in values ranging from 0-100.

^b^ Late positive potential (LPP) values are represented in amplitudes (averaged across the electrode cluster)

^c^ HC: healthy controls.

**SUPPLEMENT 5**

**Dimensional analysis with CAPE**

In order to dimensionally test for associations between CAPE scores and the fear learning indices across subjects, we included CAPE scores (mean-centred) in our mixed models with interaction terms including CS type x CAPE (for acquisition) and CS type x CAPE x phase/block (for extinction). LDS were tested by simple linear regressions, using CAPE scores as the IV. For LPPs during acquistion, LPPs across all trials were calculated for this analysis.

**Acquisition**

A significant interaction between CS type and CAPE scores was found for expectancy ratings, *b* = –7.97, *SE* = 3.02, *t*(258) = –2.64, *p* = .009. The CS Type × CAPE interaction was also significant for valence ratings, *b* = –9.11, *SE* = 3.91, *t*(258) = –2.33, *p* = .021 and arousal ratings, *b* = –11.46, *SE* = 4.18, *t*(258) = -2.74, *p* = .006. Overall, this indicated that as the CAPE scores increased, the CS discrimination decreased (i.e. less differential acquisition learning). No significant interactions were found for fear ratings, *b* = –7.37, *SE* = 3.89, *t*(258) = -1.90, *p* = .06.

**Generalization**

CAPE scores significantly predicted the LDS for expectancy ratings, *b* = –5.59, *SE* = 2.14, *t*(130) = –2.62, *p* = .010; indicating a negative association between CAPE scores and LDS (i.e. smaller LDS with increasing CAPE scores, thus more generalization). No significant associations were found for valence ratings, arousal ratings, or fear ratings (all *p*s > .13).

**Extinction**

No significant three-way interaction were found for any of the ratings (all *p*s > .8).

Overall, no significant associations between CAPE scores and LPPs and FPS were observed in any of the phases (all *p*s > .08).

**Figure S4**

*
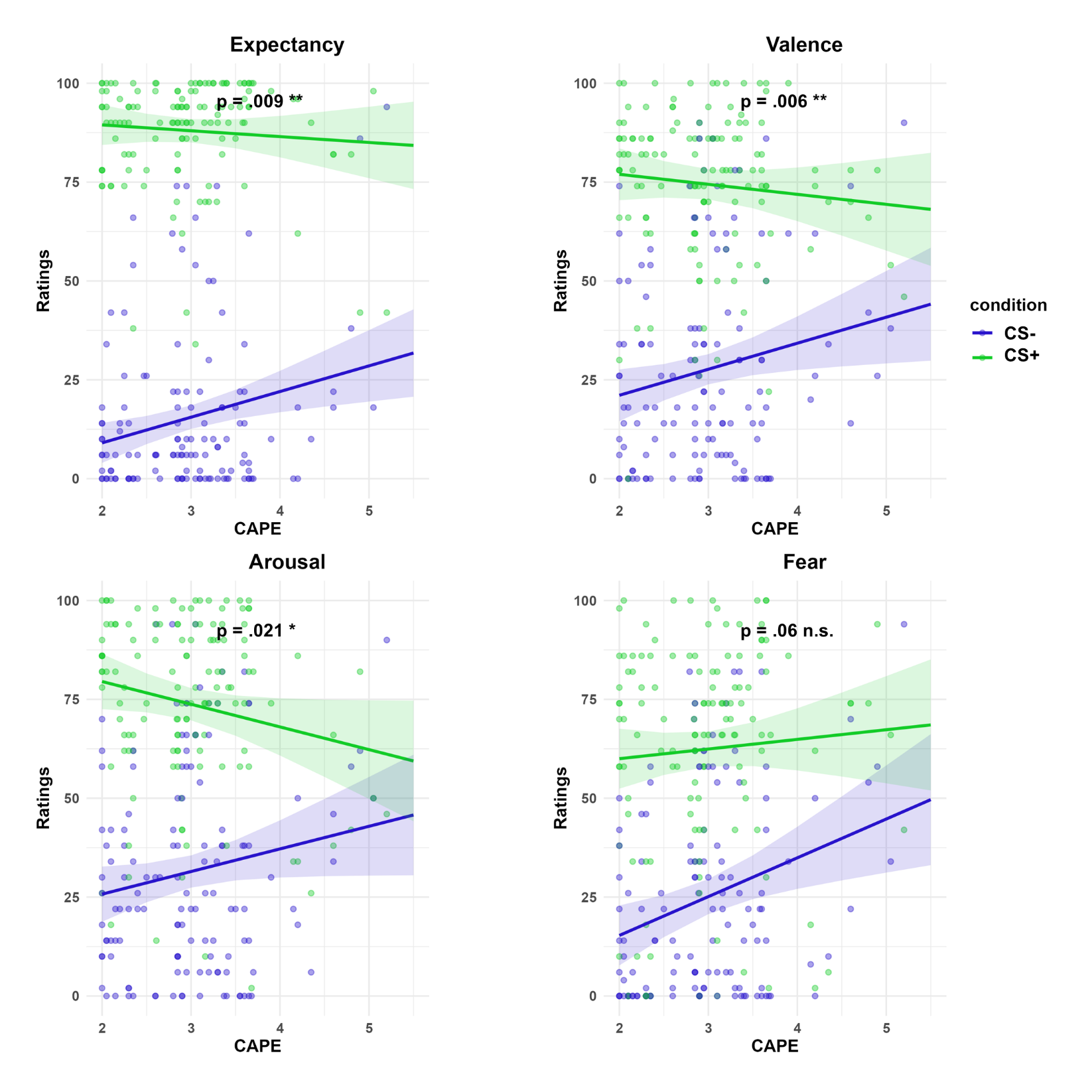
Interaction between CAPE and CS type on ratings after the acquisition phase*

*Note.* CAPE = Community Assessment of Psychic Experiences (not centered). Lines represent model-predicted values from linear mixed-effects models; shaded areas indicate 95% confidence intervals. Scatter points represent observed values. * *p* < .05 ** *p* < .01 *** *p* < .001

**

**Figure S5**

*
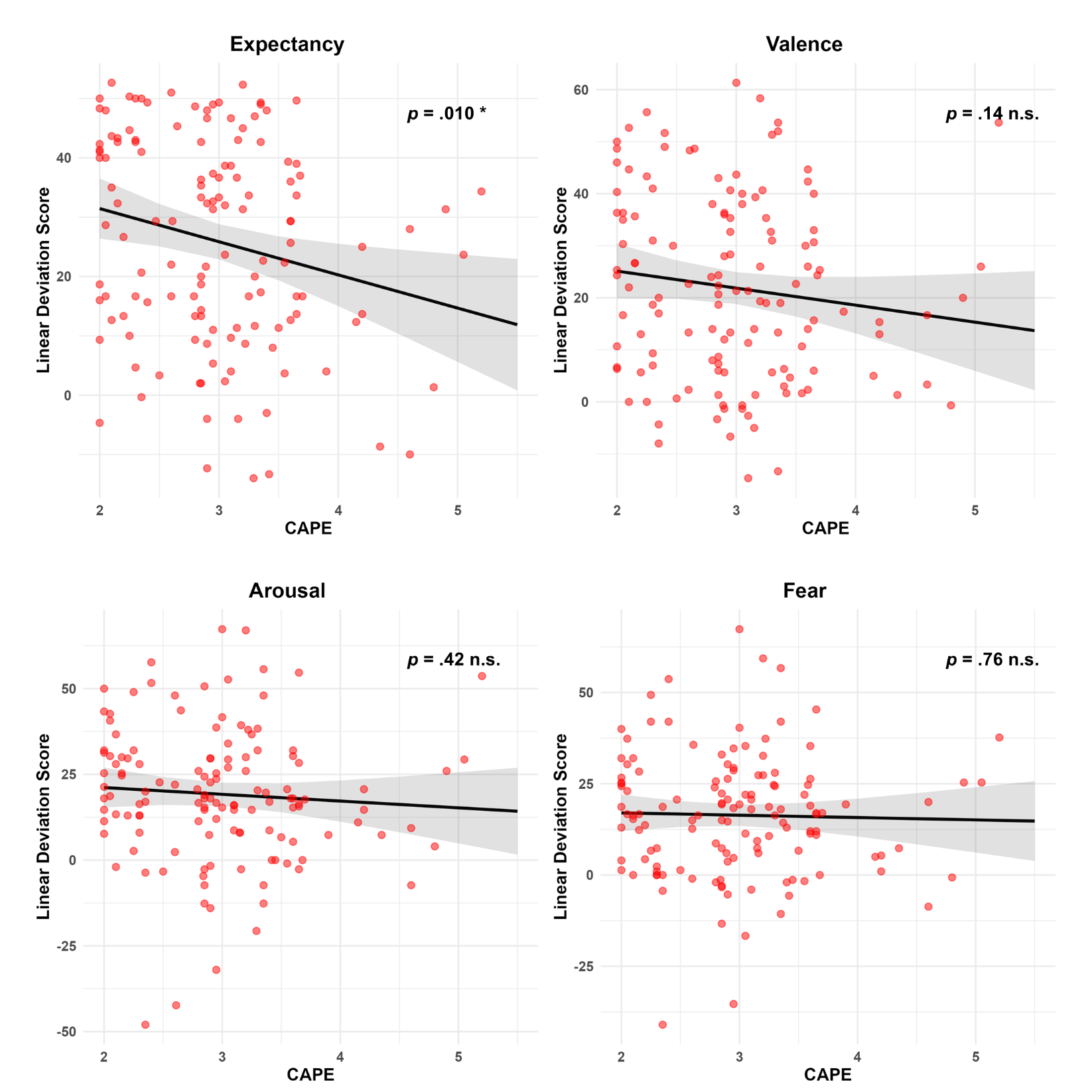
* *Association between CAPE and Linear Deviation Score in ratings after the generalization phase*

*Notes.* CAPE = Community Assessment of Psychic Experiences. Black line represents model-predicted values from linear mixed-effects models; shaded ribbon indicates 95% confidence intervals. Red dots show observed data points. * *p* < .05 ** *p* < .01 *** *p* < .001

**SUPPLEMENT 6**

**Subgroup analysis comparing PP, CHR and HC**

Subgroup analyses comparing CHR vs. PP vs. HC were conducted for each analysis, using the same models described above and adding the group predictor to each model with three levels (i.e. CHR, PP, HC). Pairwise comparisons were conducted when necessary. In order to test the group differences in generalization, a one way ANOVA was conducted with LDS as the DV and the group (three levels: CHR, PP, HC) as the IV.

**Acquisition**

A significant group × CS type interaction was found for valence ratings, with reduced CS+ vs. CS- differentiation only in the PP group compared to HCs (*b* = –17.46, *SE* = 6.21, *t*(256) = –2.81, *p* = .005). This was driven by increased responses to CS- in the PP group (*t*(258) = 2.99, *p* = .04). For fear ratings, a similar interaction was observed only between the PP group vs. HCs (*b* = –12.78, *SE* = 6.21, *t*(256) = –2.06, *p* = .041), again driven by elevated CS- responses in the PP group (*t*(258) = 3.50, *p* = .007). No significant group differences emerged for expectancy or arousal ratings (*ps* > .8). A significant group × CS type interaction was found for FPS, with reduced CS+ vs. CS- differentiation only in the PP group compared to CHR (*b* = –6.00, *SE* = 2.35, *t*(206) = –2.55, *p* = .011).

**Generalization**

A one-way ANOVA on LDS for expectancy ratings revealed a significant group effect (*F*(1, 130) = 4.90, *p* = .009), with only the PP group showing a lower LDS (i.e., stronger overgeneralization) than HCs (*t*(129) = –3.01, *p* = .009). No group differences were found for valence, arousal, or fear ratings (*ps* > .10).

**Extinction**

A significant group × CS type × phase interaction was observed for valence ratings, indicating slower extinction only in the PP group compared to HCs (*b* = 18.07, *SE* = 7.48, *t*(509) = 2.42, *p* = .016). The CS+ vs. CS- difference declined, from generalization to extinction, from 61 to 14 in HCs, and from 45.94 to 17 in the PP group. CS- ratings did not change significantly in any group (HC: *t*(256) = 0.22, *p* = 1.00; PP: *t*(256) = 1.70, *p* = .87; CHR: *t*(256) = –0.41, *p* = 1.00). CS+ ratings significantly decreased in all groups (HC: *t*(256) = –11.38, *p* < .0001; PP: *t*(256) = –9.73, *p* < .0001; CHR: *t*(256) = –7.01, *p* < .0001). The CS+ vs. CS- contrast remained significant only for the PP group (*t*(227) = 3.93, *p* = .006); it was not significant in HCs (*t*(227) = 2.77, *p* = .199) or CHR (*t*(227) = 2.57, *p* = .301).

A significant interaction was also found for arousal ratings, *b* = 16.34, *SE* = 7.12, *t*(509) = 2.29, *p* = .022, with HCs showing a greater reduction in CS+ vs. CS- difference (from 56.50 to 13.86) only compared to the PP group (from 40.09 to 17.27). CS- ratings remained unchanged (HC: *t*(247) = 0.38, *p* = 1.00; PP: *t*(247) = –0.75, *p* = 1.00; CHR: *t*(247) = 1.56, *p* = .92), while CS+ ratings decreased in all groups (HC: *t*(247) = –8.83, *p* < .0001; PP: *t*(247) = –6.71, *p* < .0001; CHR: *t*(247) = –4.53, *p* = .0006). The CS+ vs. CS- contrast remained significant for PP (*t*(234) = 4.03, *p* = .004) and CHR (*t*(234) = 3.65, *p* = .02), but not for HCs (*t*(234) = 2.78, *p* = .197).

No significant group differences emerged for expectancy or fear ratings (*ps* > .15), nor for LPPs in any phase (*ps* > .14).

**SUPPLEMENT 7**

**Demographic Variables – Differences among genders**

In order to test for differences among gender groups in the fear learning indices across subjects, we included the interaction term CS type x gender (for acquisition and generalization) and CS type x gender x phase/block (for extinction) in the mixed models. To test the differences in LDS, a one-way ANOVA was conducted with Gender as the IV.

First, we examined whether gender (regardless of at-risk status) influenced outcome measures that revealed significant group differences (i.e., at-risk vs. HC): valence ratings during the acquisition phase, expectancy ratings during generalization, and valence and arousal ratings during extinction. We found gender differences in expectancy ratings during the generalization phase, as indicated by a significant gender × CS(/GS) type interaction. Specifically, participants who identified as diverse showed a larger discrimination in ratings between GS+ and CS− compared to both men (*b* = 53.94, *t*(643) = 2.68, *SE* = 20.15, *p* = .008) and women (*b* = 57.06, *t*(643) = 2.89, *SE* = 19.74, *p* = .004). The LDS of the expectancy ratings, on the other hand, did not differ among genders, *F*(1, 130) = 0.56, *p* = .574. Valence ratings did not differ among genders during acquisition (men vs. women: *b* = 2.58, *t*(256) = .40, *SE* = 6.49, *p* = .69; men vs. diverse: *b* = 22.25, *t*(256) = 0.96, *SE* = 23.22, *p* = .34; women vs. diverse: *b* = 19.76, *t*(256) = 0.86, *SE* = 22.76, *p* = .39). Also, neither valence (men vs. women: *b* = 6.09, *t*(509) = 0.79, *SE* = 7.75, *p* = .43; men vs. diverse: *b* = 6.50, *t*(509) = 0.23, *SE* = 27.75, *p* = .82; women vs. diverse: *b* = 0.41, *t*(509) = 0.02, *SE* = 27.20, *p* = .99), nor arousal (men vs. women: *b* = 2.19, *t*(509) = 0.27, *SE* = 8.05, *p* = .79; men vs. diverse: *b* = 5.25, *t*(509) = 0.18, *SE* = 28.82, *p* = .86; women vs. diverse: *b* = 3.06, *t*(509) = 0.11, *SE* = 28.24, *p* = .91). differed among genders during extinction.

Lastly, during acquisition, women had smaller CS discrimination in FPS as compared to men on a trend level, *b* = -4.07, *t*(206) = -1.99, *SE* = 2.04, *p* = .048. No significant differences emerged for any of the other variables in any of the phases.

**SUPPLEMENT 8**

**Demographic Variables – Differences among education groups**

To examine differences among education groups in fear learning indices, we incorporated interaction terms into the mixed-effects models: CS type × education for both acquisition and generalization phases, and CS type × education × phase/block for extinction. Differences in LDS were analysed using separate one-way ANOVAs, with Education entered as the IV.

**Acquisition**

A significant education × CS type interaction emerged for expectancy ratings, showing a smaller CS+ vs. CS- discrimination in highly educated participants compared to the medium education group (*b* = –24.27, *SE* = 5.43, *t*(256) = –4.47, *p* < .001). For valence ratings, lower (*b* = 29.75, *SE* = 14.31, *t*(256) = 2.08, *p* = .039) and medium education (*b* = 19.93, *SE* = 7.11, *t*(256) = 2.80, *p* = .005) groups showed greater CS discrimination than the high education group. For arousal ratings, the medium group showed a larger CS discrimination than the high group (*b* = 21.59, *SE* = 7.63, *t*(256) = –2.83, *p* = .005). For fear ratings, both low (*b* = 29.83, *SE* = 14.06, *t*(256) = 2.12, *p* = .04) and medium (*b* = 18.54, *SE* = 6.99, *t*(256) = 2.65, *p* = .008) education levels showed greater CS discrimination than high education. No effects were found for LPPs or FPS (all *ps* > .07).

**Generalization**

For early LPPs, a significant interaction indicated that medium-educated participants showed a larger GS+ vs. CS- discrimination than highly educated ones (*b* = 1.57, *SE* = 0.63, *t*(3168) = 2.49, *p* = .013). No significant effects were found for late LPPs. LDS scores did not differ between education groups for either early (*F*(1, 130) = 1.55, *p* = .216) or late LPPs (*F*(1, 130) = 0.43, *p* = .653). For FPS, a significant interaction showed that the medium group had greater CS+ vs. CS- discrimination than the high group (*b* = 6.35, *SE* = 2.14, *t*(505) = 2.97, *p* = .003). LDS did not differ significantly (*F*(1, 104) = 1.66, *p* = .196). No group differences were found for valence, arousal, or fear ratings.

**Extinction**

For late LPPs, a significant CS type × education × phase interaction indicated that high education was associated with impaired extinction compared to low (*b* = –5.09, *SE* = 1.94, *t*(3148) = –2.62, *p* = .009). Medium education group also was associated with impaired extinction compared to low (*b* = –3.56, *SE* = 1.78, *t*(3148) = –1.99, *p* = .046). The “absolute CS discrimination ” in LPPs in the high education group increased (0.42 to 0.71), showed a minimal change in the low group (–2.86 to 2.5), and a reduction in the medium group (–1.98 to –1.06). However, within-group comparisons showed no significant change in CS+ or CS- between extinction blocks (*ps* > .09), nor any CS+ vs. CS- difference in the final block (*ps* > .98). No significant three-way interactions emerged for early LPPs (all *ps* > .097).

For FPS, a significant CS type × education × phase interaction indicated slower extinction in highly educated individuals compared to those with medium education (*b* = 5.26, *SE* = 2.13, *t*(402) = 2.46, *p* = .014). CS discrimination decreased more steeply in the medium group (7.96 to 0.91) than in the high group (1.61 to –0.25). CS+ reactivity decreased significantly for the medium group (*t*(192) = –10.73, *p* < .0001), but not for the high group (*t*(195) = –1.21, *p* = .99). No change was observed for CS- in either group (*ps* > .90), and the CS+ vs. CS- discrimination was non-significant during extinction in any of the groups (all *ps* = 1).

No significant three-way interactions were observed for expectancy or fear ratings (*ps* > .14).

# **Table S2.**

# ***Mean (M) and standard deviations (SD) of EEG Trials Remaining After Artifact Rejection***

| Phase | Stimulus | *M* | *SD* | Total Trials |
| --- | --- | --- | --- | --- |
| Acquisition | CS– (1st block)^a^ | 7.76 | 1.46 | 9 |
| Acquisition | CS+ (1st block)^a^ | 8.14 | 1.17 | 9 |
| Acquisition | CS– (2nd block) | 9.39 | 0.91 | 10 |
| Acquisition | CS+ (2nd block) | 9.69 | 0.64 | 10 |
| Acquisition | CS– (combined) ^a^ | 17.14 | 1.80 | 19 |
| Acquisition | CS+ (combined) ^a^ | 17.83 | 1.35 | 19 |
| Generalization | CS– | 11.74 | 0.50 | 12 |
| Generalization | GS– | 11.82 | 0.46 | 12 |
| Generalization | GSU | 11.66 | 0.59 | 12 |
| Generalization | GS+ | 11.65 | 0.59 | 12 |
| Generalization | CS+ | 11.68 | 0.63 | 12 |
| Extinction | CS– (1st block)^a^ | 9.84 | 0.46 | 10 |
| Extinction | CS+ (1st block)^a^ | 9.73 | 0.55 | 10 |
| Extinction | CS– (last block) | 9.66 | 0.64 | 10 |
| Extinction | CS+ (last block) | 9.70 | 0.60 | 10 |
| Extinction | CS– (combined)^b^ | 29.20 | 1.08 | 30 |
| Extinction | CS+ (combined)^b^ | 29.20 | 1.18 | 30 |

^a^ The first trial of the Acquisition phase for each CS was excluded. Therefore, the total number of trials in the first Acquisition block is 9 (out of 10) per CS, and the total number of trials for the entire Acquisition phase is 19 (out of 20) per CS.

^b^ The first trial of the Extinction phase for each CS was excluded. Therefore, the total number of trials in the first Extinction block is 10 (out of 11) per CS, and the total number of trials for the combined Extinction phase is 30 (out of 31) per CS.

**References**

Blumenthal, T.D., Cuthbert, B.N., Filion, D.L., Hackley, S., Lipp, O.V., Van Boxtel, A., 2005. Committee report: Guidelines for human startle eyeblink electromyographic studies. Psychophysiology 42(1), 1–15.

Kuhn, M., Wendt, J., Sjouwerman, R., Büchel, C., Hamm, A., Lonsdorf, T.B., 2020. The neurofunctional basis of affective startle modulation in humans: Evidence from combined facial electromyography and functional magnetic resonance imaging. Biological Psychiatry 87(6), 548–558.

Luck, S.J., 2014. An introduction to the event-related potential technique. MIT press.

Neurobehavioral Systems Inc, Presentation, Version 23.0. Berkeley, CA.

Stegmann, Y., Schiele, M.A., Schümann, D., Lonsdorf, T.B., Zwanzger, P., Romanos, M., Reif, A., Domschke, K., Deckert, J., Gamer, M., 2019. Individual differences in human fear generalization—pattern identification and implications for anxiety disorders. Translational Psychiatry 9(1), 307.

Viola, F.C., Debener, S., Thorne, J., Schneider, T.R., 2010. Using ICA for the analysis of multi-channel EEG data. Simultaneous EEG and fMRI: Recording, Analysis, and Application: Recording, Analysis, and Application, 121–133.

Winkler, I., Debener, S., Müller, K.-R., Tangermann, M., 2015. On the influence of high-pass filtering on ICA-based artifact reduction in EEG-ERP, 2015 37th Annual International Conference of the IEEE Engineering in Medicine and Biology Society (EMBC). IEEE, pp. 4101–4105.
